# Added value of MSCA doctorates: A mixed-methods study

**DOI:** 10.12688/openreseurope.23294.1

**Published:** 2026-07-02

**Authors:** Malay Bera, Michaela Bitsakis, Audrey Arfi, Laurence Marrama-Rakotoarivony, Teresa Cuartero-Lausin, Gergana Simeonova-Arida, Thomas Vyzikas, Helena Magliarelli

**Affiliations:** 1 Marie Skłodowska-Curie Doctoral Networks, European Research Executive Agency, Brussels, Belgium

**Keywords:** Marie Skłodowska-Curie Actions (MSCA), Doctoral Networks (DN), Innovative Training Networks (ITN), Doctoral education in Europe, Research policy, Impact assessment, Horizon Europe, Horizon 2020

## Abstract

This study presents the first comprehensive and robust assessment of the added value of Marie Skłodowska-Curie Actions (MSCA) Doctoral programmes funded under the Innovative Training Networks (ITN) and Doctoral Networks (DN) calls, relative to non-MSCA doctoral programmes. Drawing on a mixed-methods design combining a literature review, qualitative interviews, and a large-scale quantitative survey involving principal investigators, supervisors, and doctoral researchers (current and graduates), the study evaluates the added value and impact of MSCA ITN and DN across six core dimensions: triple “i” mobility (international, intersectoral, and interdisciplinary), training, supervision, social innovation and working conditions, career development and networking, and structuring effects on European doctoral education. Findings demonstrate that MSCA Doctoral programmes deliver a significantly higher and more integrated triple “i” mobility experience compared to non-MSCA. 90% of MSCA fellows collaborate internationally, and 88% demonstrate intersectoral mobility across academic and non-academic sectors (around three times the rate of non-MSCA fellows), broadening career trajectories beyond academia. Interdisciplinary training further enables well-rounded skill acquisition across fields. MSCA fellows report substantially higher satisfaction with transferable skills development, particularly in networking, public speaking, teamwork, and intersectoral knowledge transfer. Supervision quality is also markedly stronger, with higher rates of multidisciplinary supervisory teams and formalised supervision agreements supporting research excellence and doctoral progression. Working conditions further reinforce these outcomes, with MSCA fellows reporting greater satisfaction with their salary and MSCA supervisors expressing greater satisfaction with budgets for research, training, and mobility. Career impact is stronger as well, with 92% of MSCA fellows securing employment within one year of graduation, and one third transitioning successfully beyond academia, 3.5 times the rate among non-MSCA graduates. At the systemic level, MSCA ITN and DN programmes generate lasting structuring effects, strengthening doctoral training and research frameworks across Europe. The study recommends increasing programme visibility, raising funding levels, extending programme duration, and pursuing administrative simplification to maximise long-term impact and advance alignment with doctoral education across the European Education Area and European Research Area.

## 1. Introduction

The Marie Skłodowska-Curie Actions (MSCA) ^i^ are the European Union’s flagship programme for supporting research training, mobility, and career development for researchers at all career stages. The MSCA are implemented under the European Union’s multiannual framework programmes, ^ii^ and are managed by the European Research Executive Agency (REA) on behalf of the European Commission. The MSCA aim to strengthen Europe’s research and innovation capacity by fostering excellence, mobility, and collaboration across countries, sectors, and disciplines. Rooted in the principles of excellent science, mobility, openness to the world, inclusiveness, effective supervision, open science, and support for the European Green Deal, the MSCA promote high-quality research environments aligned with the European Charter for Researchers.

One of the key components of the MSCA under the EU’s ongoing framework programme, Horizon Europe (2021–27) is the Doctoral Networks (MSCA-DN; Innovative Training Networks (ITN) under Horizon 2020 (2014–2020)), which funds international partnerships between universities, research institutions, businesses, and other organisations to design and implement structured doctoral training and research programmes. These networks aim to train highly skilled researchers, enhance their creativity and innovation potential, and improve their long-term employability. The MSCA Doctoral Networks strengthen research excellence across Europe, as well as Europe’s doctoral training landscape, by combining cutting-edge scientific training with tailored skills development. By integrating structured mobility, personalised supervision, and exposure to non-academic sectors, the programme equips researchers with the ability to translate ideas into impact, bridging the gap between academic knowledge and real-world innovation. Mobility (international, intersectoral, and interdisciplinary) lies at the heart of the MSCA Doctoral Networks, and this is where the programme’s added value truly emerges. Cross-border and cross-sector exchanges allow doctoral candidates to work with leading experts, access state-of-the-art facilities, and develop highly adaptable mindsets. Such mobility accelerates knowledge circulation and fosters a research culture that is open, collaborative, and internationally competitive. The integration of industry, SMEs, NGOs, public bodies, and cultural institutions into doctoral training ensures that research outputs are connected to societal needs, and that researchers gain hands-on insight into how science drives policy, innovation, and economic growth. This intersectoral dimension is a core added value that few other doctoral programmes consistently deliver. For participating organisations, the MSCA Doctoral Networks act as catalysts for strengthening long-term collaborations, securing access to diverse talent, and expanding research capabilities. The programme encourages institutions to rethink supervision, design structured doctoral curricula and build sustainable partnerships that continue well beyond the project’s duration.

The MSCA DNs are implemented through three different types of actions: Standard Doctoral Networks, Industrial Doctorates (which bridge academia and industry), and Joint Doctorates (which lead to joint or multiple doctoral degrees awarded by participating institutions). The MSCA DN projects typically run for four years, involving 10 beneficiaries and 6 associated partners on average. These consortia usually recruit up to 15 Doctoral Candidates for a maximum of 36 month-fellowship (or 48 month-fellowship for more recent Joint Doctorates). The MSCA Doctoral Networks calls for proposals are highly competitive, as demonstrated by the high number of applications submitted every year (more than 1300 submitted proposals on average) and a low success rate, ranging from 7–8% to 15% depending on the year and the scientific domain. Since the beginning of Horizon Europe (2021), 599 HE-DN project grants have been signed as of 2025, out of which 85% were standard Doctoral Networks, 8.3% were Industrial Doctorates and 6.5% were Joint Doctorates. The MSCA DN budget for HE represents a total budget of 3.3 billion EUR, which represents approximately half of the total Marie Sklodowska-Curie Actions (MSCA) programme budget over the multi-annual financial framework. As of December 2025, more than 4500 doctoral candidates have been recruited and trained within the HE-DN projects which continues to increase as DN 2024 recruitments are ongoing.

Analysing the added value of an EU research and doctoral funding programme is essential to demonstrate the benefits arising specifically from action at European level and that individual countries or institutions could not deliver alone. Such assessment supports transparency and accountability in the use of public funds and provides an evidence base for improving future programme design and implementation. It also clarifies how the programme contributes to key EU priorities, including the strengthening of the European Research Area, the promotion of researcher mobility, and the fostering of interdisciplinary ^iii^ and intersectoral ^iv^ cooperation. In addition, understanding the programme’s added value helps participating organisations enhance their practices, increases the visibility of achievements, and illustrate the long-term impact for researchers, institutions, and society, thereby supporting a more competitive, innovative, and cohesive European research ecosystem.

Several studies on doctoral training in Europe have been carried out previously, and the European Commission also periodically publishes reports on the impact of the MSCA programme. ^1– 8^ The present study builds on the insights from previous studies through the first ever comprehensive and robust systematic assessment of the added values of the MSCA focussing on the Innovative Training Networks (ITN) and the Doctoral Networks (DN). The study incorporates both qualitative and quantitative aspects based on literature review, interviews (with MSCA and non-MSCA stakeholders), and a large-scale survey (including MSCA and non-MSCA supervisors/Principal Investigators (PIs) and fellows) with a very wide global coverage ( Figure 1). The findings of the study present a comprehensive and well-grounded overview of the added values of the MSCA doctorates by drawing from the responses of the MSCA participants. These results are further strengthened through comparison with feedback from non-MSCA participants, ensuring a balanced and reliable assessment. The study’s robustness is supported by exceptionally high survey participation, with responses from individuals involved in 85% of all ITN and DN projects funded between 2014 and 2024.

**Figure 1.**
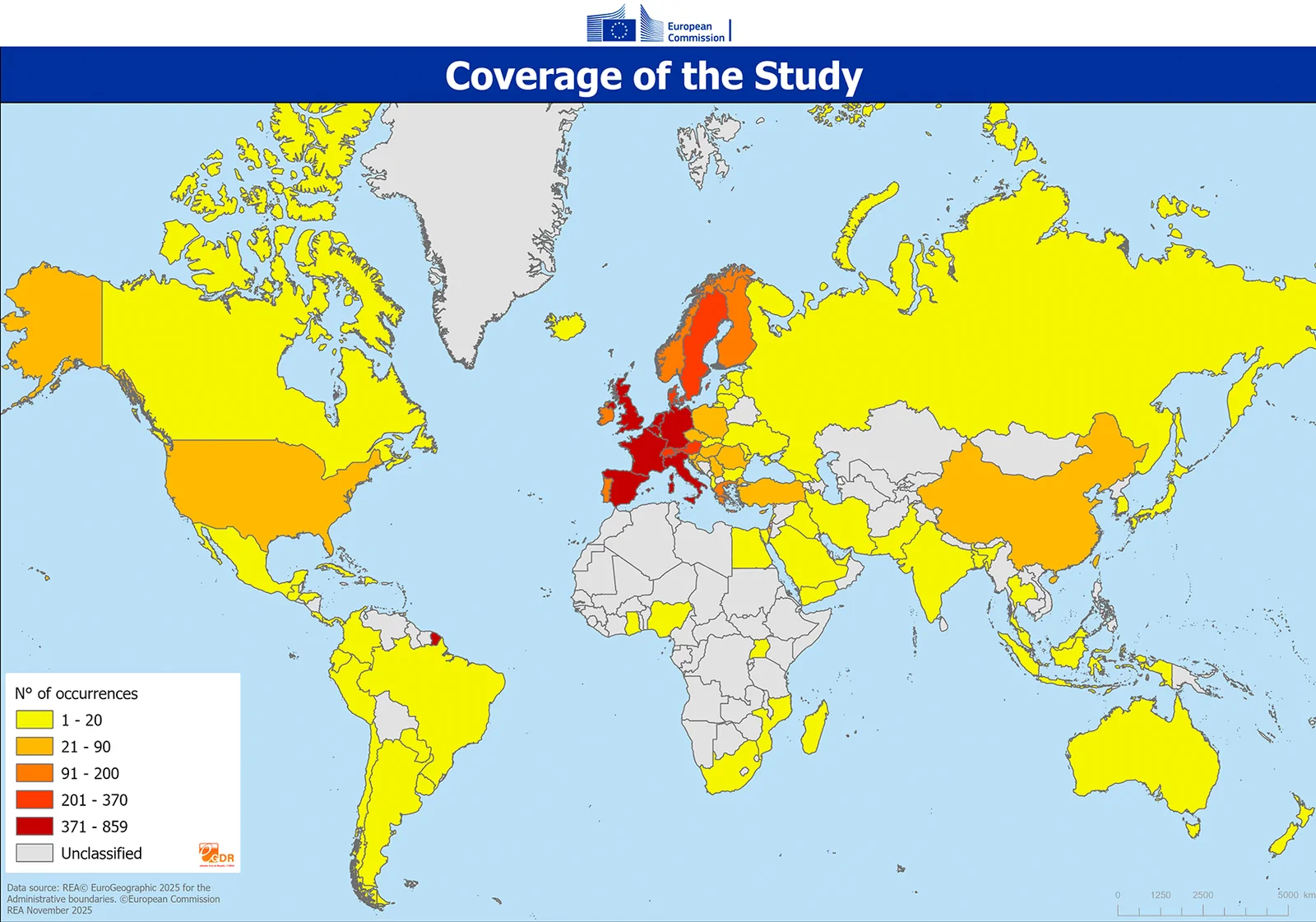
Coverage of the study.

Comparatively analysing the MSCA participants’ responses in relation to the non-MSCA participants in the study reaffirms that the MSCA ITN and DN consistently distinguish themselves as research frameworks of high international standard.

## 2. Methodology

The study had three main components: (1) a literature review, (2) interviews, and (3) a survey (which had both quantitative and qualitative aspects). Based on a review of 110 existing literature items, questions were prepared for the interviews and the survey to assess the added values of the MSCA ITN and DN in relation to several key themes or indicators. These indicators were identified based on the objectives and key principles of the MSCA, and their relevance for the study was confirmed through the literature review. These key themes include: (1) international mobility, (2) intersectoral mobility, (3) interdisciplinary mobility, (4) training, (5) supervision, (6) working conditions, (7) career development and networking, and (8) structuring effects. These key areas served as the points of focus which helped design the questions for the interviews and the survey to rigorously validate previous findings, trace their evolution over time, and identify any emerging trends.

Prior to launching the survey, 34 semi-structured interviews were conducted with key stakeholders including MSCA and non-MSCA supervisors (and Principal Investigators) ^v^ and fellows, project managers, and other international stakeholders in doctoral education and training. Although the sample size for the interviews limits generalisability, the qualitative data served as an essential exploratory tool to identify relevant themes, refine the survey design, and ensure strong contextual alignment to enhance the relevance and practical value of the present work for all stakeholders.

Participants’ well-being was recognised and addressed in the design of the study. Since all participants were stakeholders in doctoral research and training, the questions for the survey as well as interviews were designed to gather information on the state of play of doctoral training in Europe to provide even better facilities, resources, working conditions, and opportunities to future researchers. The survey questionnaire was specifically designed to hear the voices of the participants. Most of the questions offered participants the opportunity to provide subjective open-ended opinions in addition to selecting pre-identified options. Considering the well-being of both MSCA and non-MSCA participants, the questionnaire also aimed to identify which aspects of MSCA can enhance non-MSCA doctoral experiences and vice versa. Both open-ended and closed responses have been taken into account to analyse the impact and added value of the MSCA DN, and to identify challenges and provide future recommendations.

To conform with the ethics of research involving human participants, all participants of the study (both interview and survey) have been anonymised in the report in compliance with General Data Protection Regulation (GDPR) ^9^ to maintain participant confidentiality. Participation in the study was completely voluntary, and participants had the right to withdraw at any time without justification. All participants in both components of the study were provided prior information regarding the purpose of the study. A Data Protection Notice (DPN) ^10^ was shared with the participants demonstrating ethical commitment to maintain their anonymity. The DPN also included sufficient information on the procedure to withdraw, should any participant choose to do so. Additionally, written consent was obtained from the interview participants for recording the video interviews and processing relevant information after deidentification of personal data for the purpose of the study. The data collected through survey and interview were stored securely in accordance with the data protection policy communicated to the participants and have been used only for the purposes of the study. The data obtained have been processed with appropriate research ethics and the wellbeing of the participants in mind and have not been used for any other purpose than the current study assessing the state of play of doctoral research in Europe to provide recommendations for future enhancement of MSCA Doctoral Networks.

The survey was sent to a mailing list of approximately 58,437 individuals identified from the MSCA database comprising of individuals with connection to MSCA ITN/DN and PF/IF beneficiaries who were requested to circulate it further, in particular to non-MSCA potential respondents. A total of 6789 responses were received from the two main target groups: (1) Supervisors and PIs, and (2) PhD fellows (both PhD graduates and ongoing PhDs). Both the categories included individuals with experience in MSCA and Non-MSCA PhD programmes. After cleaning the raw data to eliminate incomplete submissions, 6723 responses were considered for further analysis, out of which 3191 (68% MSCA, 32% non-MSCA) were supervisors and 3532 (83% MSCA, 17% non-MSCA) were PhD graduates or ongoing PhD fellows ( Figure 2).

**Figure 2.**
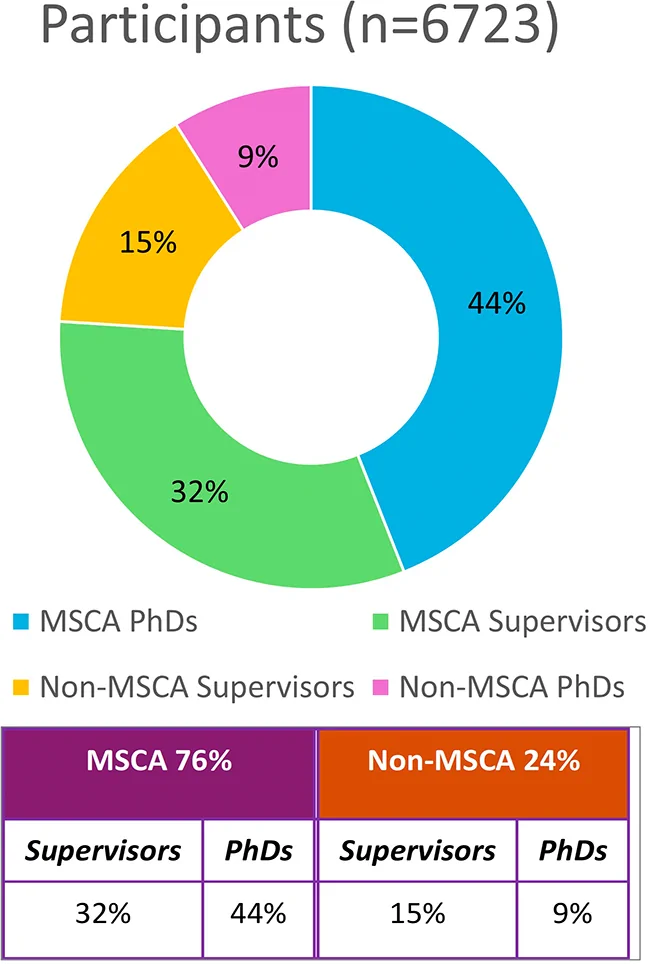
Participants of the survey.

In total, 76% of the participants represent individuals with ITN/DN experience (between 2014 and 2024) whereas only 24% of them represent non-MSCA supervisors and PhD fellows ( Figure 2). Admittedly, there is a big relative difference between the number of MSCA and non-MSCA participants, nevertheless, it cannot be discounted that non-MSCA participation comes from a large number of individuals as well (1031 supervisors and 607 PhD fellows) which can be considered as a representative sample.

As with any large-scale study, some of the methodological limitations must be acknowledged here. While the interviews provided highly valuable insights, the sample size for the in-depth interviews was limited, potentially reducing the generalisability of the opinions expressed in each group. However, it allowed the comparison between the groups and helped verify the quantitative outcomes from the survey. The survey’s methodological limitations are notable as well. For instance, respondents may not be representative of the entire population of PhD researchers and supervisors/coordinators. In particular, because of the way selected to contact them (through their peers), a part of the participants in non-MSCA funded projects might be more similar to the participants in MSCA funded projects than the general population of researchers that did not participate in any MSCA funded projects. Social desirability bias must be recognised as well, i.e., respondents may provide answers that they think are expected or desirable, rather than their true opinions. Another aspect to be mindful of is the non-response bias which is much harder to eliminate. The survey succeeded in collecting a large number of replies, including 85% coverage within the MSCA-funded projects, but it was not possible to reach all eligible respondents, particularly in the non-MSCA funded group where comprehensive census data are not available. However, despite these limitations, the survey provides valuable comparative perspectives to gather insights into the added value of MSCA-funded PhDs and to inform future funding decisions and policy developments.

## 3. Literature Review

A review of the existing literature confirmed the key areas of focus for this study, derived from the major underlying principles of the MSCA: (1) triple “i” mobility (international, intersectoral and interdisciplinary), (2) Training, (3) supervision, (4) Social innovation and working environment, (5) career perspectives and networking opportunities, and (6) structuring effects. Sources consulted during literature review include European Commission reports, and external research studies on doctoral education between 2011 and 2025. The main findings of the literature review are summarised below highlighting the performance of the MSCA with respect to the principles mentioned above, as well as the evolution of the programme and its impacts. The literature review helped identify the gaps in existing literature and established the basis for the design of the interviews and the survey employed in this study.

### 3.1 Triple “I” Mobility: International, Intersectoral, Interdisciplinary

The MSCA are known to have been the most international component of Horizon 2020, ^11^ and the MSCA ITN and DN have played a pivotal role in advancing international mobility and collaboration. The programmes effectively enable doctoral candidates to gain international experience, broadening their perspectives and horizons of excellent training, and enhancing their skills, while helping them build professional networks for career advancement. ^12^ The MSCA ITN and DN have also significantly contributed to retaining researchers in Europe, attracting global talent, and fostering international cooperation. ^11^ The MSCA ITN and DN are recognised for establishing sustainable partnerships and long-term collaborations that enhance both the visibility and reputation of European research institutions worldwide. ^11^ The MSCA contribute to international mobility not only at individual level but also fosters structured and systematic collaborations between international organisations. A previous 2023 report from the European Commission highlights that the MSCA have already strongly contributed to cross-border and international mobility of researchers by bringing together organisations based in over 139 countries worldwide. ^11^ Overall, the ITN and DN within the MSCA stand as pivotal frameworks that not only facilitate international mobility but also create robust networks of collaboration, enriching the European Research Area and contributing to a more integrated and innovative global research community.

The MSCA enhance industry-relevant skills and networking by encouraging collaboration across sectors, leading to transformative career shifts for the fellows. ^11– 15^ According to a 2020 report, non-academic sector participation in the MSCA under FP7 accounted for approximately 24%, ^16^ which increased to one-third under H2020, with the ITN representing the highest (41%) non-academic sector participation in the MSCA under H2020. ^17^ Although exposure to non-academic sector is not obligatory under the MSCA ITN/DN, it’s encouragement to facilitate intersectoral mobility has yielded highly effective results. A 2023 study on the innovative dimensions of the MSCA ITNs based on over 100 innovation-intensive projects reveals that 69% of high-capacity innovators are SMEs, demonstrating a clear correlation between industry participation (particularly SME engagement) and innovation outcomes. ^7^ According to the statistics extracted from the CORDA database, 50.38% of ITN fellows under H2020 benefited from intersectoral mobility, 16% of whom were directly recruited by the non-academic sector. Under the currently ongoing framework programme, Horizon Europe, 40% of MSCA DN fellows have had non-academic sector experience (15% directly recruited by non-academic sector) which is expected to increase as projects are still ongoing and secondments usually take place during the second reporting period (Source: CORDA 2025). ^vi^ The present study will specifically assess the latest figures related to ITN and DN to understand the intersectoral mobility of doctoral fellows and its added value compared to non-MSCA. Several studies have emphasised that the MSCA participants hosted in businesses benefit from stronger exposure to the non-academic sector, enhanced employability, and increased intersectoral mobility, with some suggesting positive effects on long-term career development and entrepreneurial activities. ^2, 11, 12, 18^ However, the participation in intersectoral mobility schemes varies, with lower involvement in some regions, particularly Central and Eastern Europe. ^19^ Further, logistical issues like visa problems, accommodation, and integration into industry settings have been recognised as possible hindrances in ensuring the effectiveness of secondments. ^2^

The League of European Research Universities (LERU) highly recommends interdisciplinarity as a good practice in research. ^20^ However, interviews with key stakeholders in the PhD labour market (e.g., Eurodoc, EUA-CDE, MCAA, PRIDE Network, LERU, ISE, Technopolis Group Belgium, EuroScience, YAE and ERA Governance) have brought forth that current doctoral training modules in general may not be putting enough value on interdisciplinarity. ^5^ Contrary to many national PhD programmes, the MSCA ITNs and DNs realise interdisciplinary research with their emphasis on bottom-up approach to funding research, and integrating multiple disciplines. ^6^ As the LERU’s 23 member organisations acknowledge, the “ITNs are interdisciplinary and instrumental in training a new generation of interdisciplinary scientists and professionals”. ^6^ Especially, the presence of business organisations in ITN and DN contributes immensely to interdisciplinary training. According to the figures published by the European Commission in 2019, about 69% of ITN fellows involved in projects with non-academic organisations engage in interdisciplinary research, compared to 36% of fellows in projects with no such organisations. ^21^ Although definitive statistics on the latest rate of interdisciplinarity in all ITNs is hard to find, studies indicate that interdisciplinarity is one of the key features of the ITNs. A 2021 study based on the experience of over 80 ITN fellows from nine ITN projects highlighted that over 80% of these ITN fellows benefit from interdisciplinarity. ^22^ Some studies have also highlighted that it would be beneficial to promote better organisation of interdisciplinary collaborations, and better align the fellows’ secondments with their PhD research. ^4^

Premised upon the findings of previous studies and reports presented above, the present study will assess the trends and impacts of the triple “i” mobility (international, intersectoral, and interdisciplinarity) with a focus on ITN and DN to offer future recommendations.

### 3.2 Training

Although historically, there has been a gap in supporting comprehensive skills development among PhD holders in general (not necessarily MSCA participants), ^23^ the MSCA, especially the ITNs are recognised for playing a significant role in enhancing employable skills. ^24^ The MSCA are recognised for setting standards for quality training at the system level through synergies, complementarity, and completeness of training, emphasising the importance of maintaining well-rounded high standards in doctoral education. ^25^ The MSCA’s focus on developing transferable skills is one of its outstanding aspects especially considering the MSCA ITN/DN’s particular emphasis on innovation and entrepreneurship. The MSCA ITN/DNs stand out with their emphasis on transferable skills training, especially considering that only one-third of PhD fellows in general in the EU receive transferable skills training, while the majority primarily focus on skills related to core research activities. ^26^ The need for transferable skills training for researchers is also acknowledged by the report on labour market perspectives for PhD graduates in Europe. ^5^ The present study will assess the success of the MSCA ITN and DNs in realising their goal to promote transferable and soft skills training while also examining whether new needs have emerged.

### 3.3 Supervision/Co-Supervision/Mentoring

The organisations participating in the ITNs and DNs adhere to the Principles of Innovative Doctoral Training and the MSCA Supervision Guidelines, deeply rooted in the European Charter for Researchers which ensures fair, transparent, and inclusive practices, promoting research excellence, stronger supervision and effective mentoring and career guidance. ^27^ According to a 2017 report, 72% of ITN fellows rated the quality of supervision as very good. ^28^ A recent trend also highlights the importance the MSCA have placed on supervisory networks, ^2^ or joint supervision frameworks setting up a supervisory team for each fellow, customised according to the requirements of the modes of actions (standard, industrial etc.), the research topic (often pluri-disciplinary) and the environment. ^2^ Substantiating the already established appraisals of supervisors among the doctoral candidates in Europe, ^29^ the most recent MSCA fellowship questionnaires confirm a very high satisfaction rate and even show an increasing trend. Although latest figures specific to the ITN and DN are hard to find, the scientific supervision has been rated “Good” or “Very good” by 83.7% and 85% of the MSCA fellows across all actions in 2021 and 2023, respectively. ^14, 30^ Although supervisors were appraised quite positively, ^8^ supervision arrangements, in the past, have not always been formalised or well communicated to the students giving rise to future complications. ^29^ The MSCA Supervision Guidelines seek to address this issue by explicitly laying down models for excellent supervision practices. The present study will assess the MSCA ITN and DN’s success in the area of supervision, understand its added values as well as limitations and offer recommendations.

### 3.4 Social Innovation/Working Environment/Employment Conditions

Doctoral fellows in many countries feel discouraged to take parental leave due to concerns about career impacts. ^4^ Yet, many researchers experience career interruptions, especially female researchers due to maternity. The MSCA address this and other challenges by promoting smooth career restart after breaks and offering various allowances such as family allowances, special needs allowances for individuals with disabilities, ^vii^ long-term leave allowances, ^viii^ the possibility of part-time work for family and personal reasons, parental leave with the possibility to extend the fellowship etc. ^12, 21^ Consequently, the MSCA fellows have historically demonstrated an ability to resume their research careers smoothly after interruptions. ^18^

In terms of recruitment policies, researchers in EU higher education institutions (HEI) have previously expressed concerns about issues related to openness, transparency, and merit-based approaches. ^31^ A 2017 assessment by DG EAC found that MSCA may not necessarily have direct immediate impact on HR management practices and processes in business organisations. ^32^ But the same study also highlighted that repeated participation in the MSCA projects helps create lasting structuring impact on the companies’ HR and recruitment strategy, helping develop stronger support structures for incoming international researchers. Recent trends indicate that most MSCA fellows feel well-integrated within their host institutions, with positive feedback on the support received. ^14, 30^ Despite this progress, awareness of the European Charter for Researchers and the Code of Conduct for recruitment remains limited, particularly among early-career researchers. ^26^ This study will examine these aspects of the working environment, employment conditions, and social innovation to assess the evolution and impact of MSCA ITN and DN in recruiting doctoral researchers and enhancing their work experience.

### 3.5 Career Perspectives and Networking Opportunities

The MSCA has been at the forefront of supporting intergenerational educational mobility ^33^^,^ ^ix^ through higher education by training generations of doctoral researchers. According to a 2018 study, a significant proportion of MSCA doctorates experience upward social mobility in terms of education compared to their parents, with a higher education level achieved by 96% compared to their mothers, and 92% compared to their fathers. ^4^ Recent reports show that a majority of the MSCA participants have considered their MSCA experience as useful or very useful for their employability, and career development and progression. ^7, 13^ In a 2022 report by DG EAC, around 60% of all MSCA fellows indicated that it would have taken them more time to smoothly proceed to their subsequent career stage without the MSCA experience, and an additional 12% of the fellows acknowledged that they would not have attained the subsequent career stage at all without the MSCA support. ^12^ In a 2023 assessment by the DG RTD, 87% of the MSCA fellows reported that the fellowship had a high or somewhat high impact on their career progression. ^11^ Based on the report of the follow-up questionnaire two-years after the completion of PhD, another 2023 study showed that 75% of the MSCA fellows found employment after PhD (42% right after completion) while 12% remained engaged in education and training activities. ^7^ The MSCA are also interconnected with other EU programmes in the career progression of researchers. For instance, the ERC plays a significant role in shaping the career trajectory of MSCA fellows who decide to continue in academia. ^34^ Although the MSCA programme’s impact on career development is generally seen positively, nearly 15% of the respondents (including ongoing fellows) in a 2022 report expressed uncertainty about the impact of the MSCA programme on their career development and progression. ^13^ The fellows have expressed concerns about the lack of available positions post-MSCA, leading to intense competition for opportunities in the job market. ^13^ With this context of the evolving nature of the job market in the background, the present study will also analyse the latest trends in the MSCA’s impact on the employability of ITN and DN fellows after the completion of their PhD.

### 3.6 MSCA Structuring Effect

The MSCA Doctoral Networks aim to foster excellence by structuring doctoral research and training in Europe and beyond, setting up sustainable research frameworks and good practices in research institutions, while positively reshaping the research landscape in Europe. ^7^ The local training capacities of the participating organisations benefited by aligning to the highest standards facilitated by the consortium structure of the MSCA projects. The ITNs have contributed to the structuring effects of the MSCA by promoting the principles of Innovative Doctoral Training (IDT), ^35^ and spreading the introduction of industry-relevant training into institutional curricula. ^11^ The reference documents like the European Charter for Researchers, the Code of Conduct for the Recruitment of Researchers, and MSCA Supervision Guidelines help spread more transparent recruitment and appointment procedures, and foster the adoption of fairer working conditions for researchers embedded in the MSCA programme. ^5, 25, 34, 36^ In a similar way, the MSCA Green Charter is known to contribute to spreading awareness of environmental sustainability in the scientific community and scientific work, encouraging researchers to identify good practices likely to minimise the negative environmental impact of their research activities and to consider implementing them in their projects. Furthermore, as the study evaluating Excellent Science in the European Framework Programmes for Research and Innovation (Horizon 2020) suggests, the resources offered by ITN made possible the revolutionary adoption of open science principles. ^11^

Due to their nature, the MSCA joint doctoral programmes (EJD/JDs) also contribute to reforming the universities’ systems by pushing forward the harmonisation and standardisation of doctoral programmes across universities. ^11, 37^ This impact is not only limited to the institutions benefiting from the MSCA funds. Due to the high competition in securing MSCA funding, ITNs have become a *de facto* “quality label.” The MSCA programmes set the example of best practice extending their impact on doctoral programmes not only within a given university but also by helping to improve doctoral programmes at the national level, thereby, progressively advancing the university systems. ^11, 25, 28, 36, 37^

Building on these findings in the literature review, this study will delve into the impacts and added values of the MSCA ITN and DN, examining both their successes and challenges. By providing a detailed assessment, this study aims to offer insightful recommendations for future improvements, ensuring that MSCA programmes not only sustain their high levels of delivery against their objectives but also effectively address existing and emerging issues related to mobility, funding, and administrative support.

## 4. Findings: Added Value Analysis

This section summarises the results of the study on the added value of some key features of the MSCA ITN and DN in comparison to non-MSCA PhD programmes. The analysis highlights the roles and impacts of the MSCA on international mobility, intersectoral mobility, interdisciplinary mobility, training, supervision, working conditions, and career development of ITN/DN fellows compared to non-MSCA fellows. Additionally, it explores the structural effects these MSCA characteristics have on doctoral programmes across Europe.

### 4.1 Triple “I” Mobility: International, Intersectoral, Interdisciplinary

*4.1.1 Findings on the Triple “I” Mobility*

“The international networking is amazing! Many of these PhDs will form the next generation of research leaders and the groundwork for their professional network is already formed through MSCA programme.” – MSCA Supervisor (Survey).

International mobility emerges as a cornerstone of the MSCA doctoral networks, offering PhD fellows the invaluable opportunity to immerse themselves in new cultural and academic landscapes. This mobility not only enriches their research experience but also fosters robust international collaborations, essential in today’s interconnected world. The survey highlights that 17% more MSCA doctoral fellows engage in international collaborations than their non-MSCA counterparts, with MSCA supervisors also demonstrating an 11% higher rate of cross-border cooperation ( Figure 3). This emphasis on global engagement reflects the programme’s strategic orientation and its aim to foster research excellence across national boundaries. As noted by a project manager interviewed for the study, international mobility offers substantial benefits for those keen on forming European or international networks, significantly strengthening institutional collaboration across borders:

**Figure 3.**
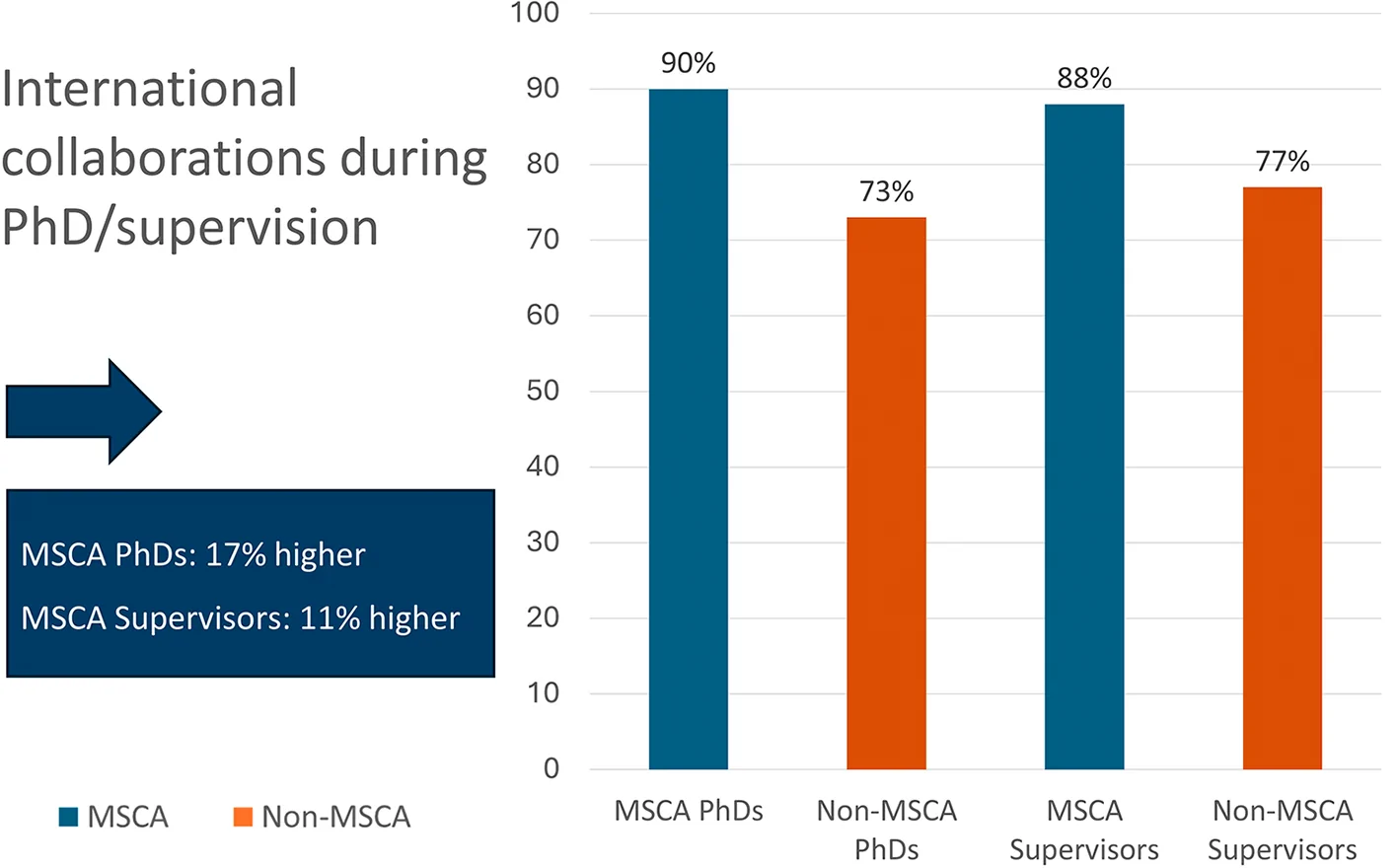
Participants who gain international experience during PhD/supervision.

“[International mobility] gives you so much more if you’re interested in having European or international network and to strengthen your collaboration with other institutions outside of your own country, that is a huge advantage.” – Project manager (Interview).

For many non-MSCA PhD fellows, however, international mobility remains a formidable challenge, often requiring them to secure personal funding to make these collaborations possible. One stakeholder insightfully observed that while national PhD programmes offer mobility opportunities, they lack integrated structures for funding, necessitating extra effort from candidates. This underscores the need for policymakers to address these disparities and consider integrating more robust support mechanisms within national frameworks to ensure equal access to the global stage. By aligning national policies with the dynamic opportunities offered by the MSCA, countries can foster a more inclusive and internationally competitive research environment.

The survey reveals a striking disparity between MSCA and non-MSCA participants in their engagement with non-academic organisations, highlighting the remarkable intersectoral experience acquired by MSCA fellows. An impressive 88% of ITN/DN fellows gain intersectoral experience, marking a 59% increase over their non-MSCA peers ( Figure 4). This robust engagement is integral to the MSCA model, where industrial PhDs inherently benefit from diverse sector exposure. But even when excluding the industrial MSCA EID/ID fellows, the intersectoral experience of MSCA non-industrial fellows stands at 65%, a substantial 36% higher than that of non-MSCA counterparts.

**Figure 4.**
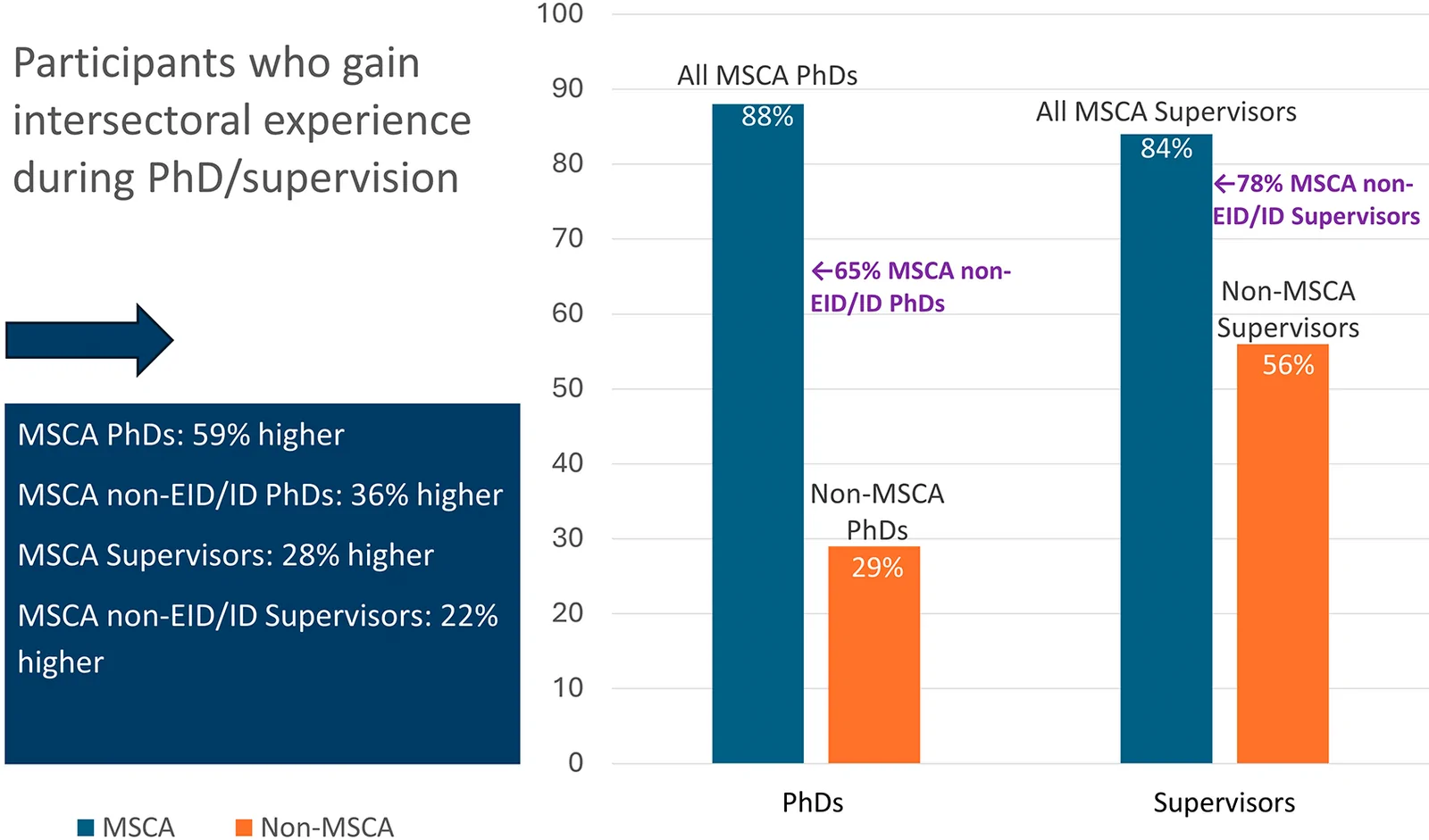
Participants who gain intersectoral experience during PhD/supervision.

Moreover, MSCA supervisors exhibit a similar trend, with 84% engaging in intersectoral collaborations, outpacing non-MSCA supervisors by 28% ( Figure 4). This isn’t simply due to the industrial supervisors, as even within the non-industrial sample, MSCA supervisors demonstrate a 22% higher rate of cross-sector engagement than their non-MSCA counterparts. As one MSCA supervisor eloquently stated, the intersectoral dimension (and triple “i” mobility as a whole) of MSCA ITN/DN programmes fosters the emergence of excellently trained young scientists, poised to bridge the gap between industry and academia with their diverse skill sets. Not only intersectorality, but all aspects of triple “i” mobility and comprehensive training in the MSCA PhD programme complement each other positively which has been felt by many participants. As one MSCA supervisor notes in the survey:

“Beyond financial support, the MSCA provides comprehensive training, networking, and mobility opportunities that are rarely matched by other funding programmes. The interdisciplinary and intersectoral collaborations encouraged by MSCA projects give researchers exposure to diverse perspectives and skills, enhancing both personal and professional growth. The international focus of MSCA funding makes researchers more adaptable and better prepared for global academic or industry careers.” – MSCA Supervisor (Survey).

These findings highlight the strategic importance of fostering intersectoral collaborations within doctoral programmes and their significant impact on innovation ecosystems. It has been highlighted by a 2023 study which shows that MSCA ITNs have demonstrated an innovation-generating capacity comparable to that of other H2020 programmes, indicating that the innovation output of ITN fellows is on par with that achieved in Research and Innovation Actions (RIA) and Innovation Actions (IA), which are typically led by more established researchers in academic and non-academic organisations. ^7^ The success of the MSCA ITN (and DN) in producing doctorates skilled in both academic and non-academic sectors offers an exemplary model for integrating intersectoral dimensions into national doctoral training frameworks. Increasing intersectorality in PhD programmes across member states can better equip scientists with the versatile capabilities needed to drive forward-thinking solutions in both industrial and academic realms. The MSCA’s approach exemplifies how multifaceted PhD programmes can be structured across Europe, enhancing expertise and driving policy dialogues toward a more inclusive and dynamic research environment within the European Research Area (ERA).

The study reveals a significantly compelling trend: MSCA ITN/DN fellows are over three times more likely to secure employment in the non-academic sector after completing their PhDs compared to their non-MSCA counterparts, among whom a substantial 90% remain in academia ( Figure 5). This enhanced mobility towards non-academic employment is a significant advantage of the MSCA DN programmes, particularly given that the majority of research-related jobs in Europe reside outside traditional academic environments. ^38^ Consequently, MSCA DNs play a vital role in preparing researchers with the diverse skill sets necessary for various career paths in research and innovation, aligning closely with global labour market dynamics.

**Figure 5.**
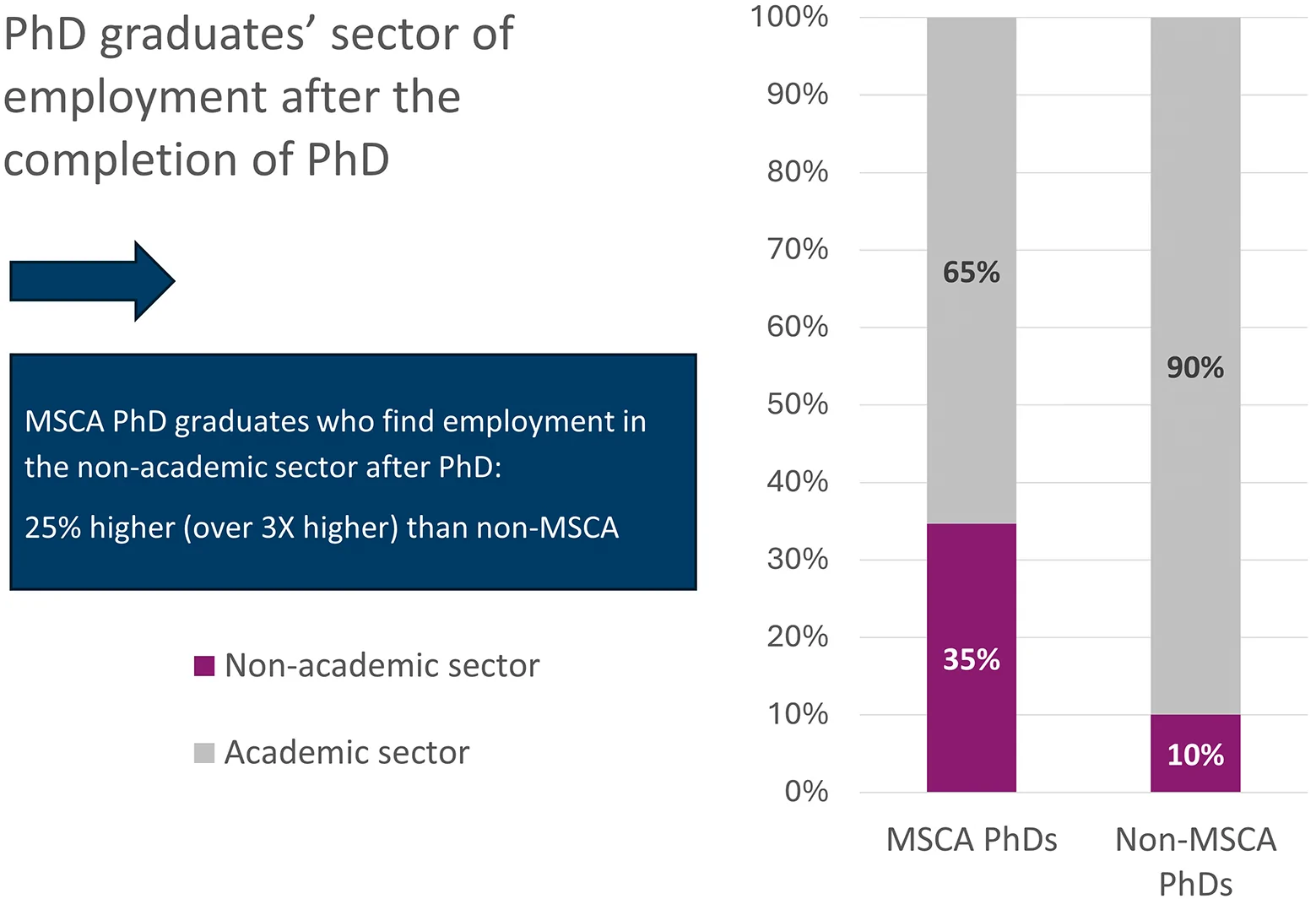
Sector of employment after the completion of PhD.

Additionally, the MSCA’s bottom-up approach significantly fosters interdisciplinarity within its funded projects. As illustrated in Figure 6, both MSCA PhDs and supervisors exhibit a 9% higher engagement in interdisciplinary research compared to their non-MSCA peers. This focus on cross-disciplinary collaboration not only broadens the scope of research but also fuels innovative solutions, ensuring that MSCA graduates are well-equipped to address complex, multifaceted challenges in an ever-evolving scientific and professional horizon.

**Figure 6.**
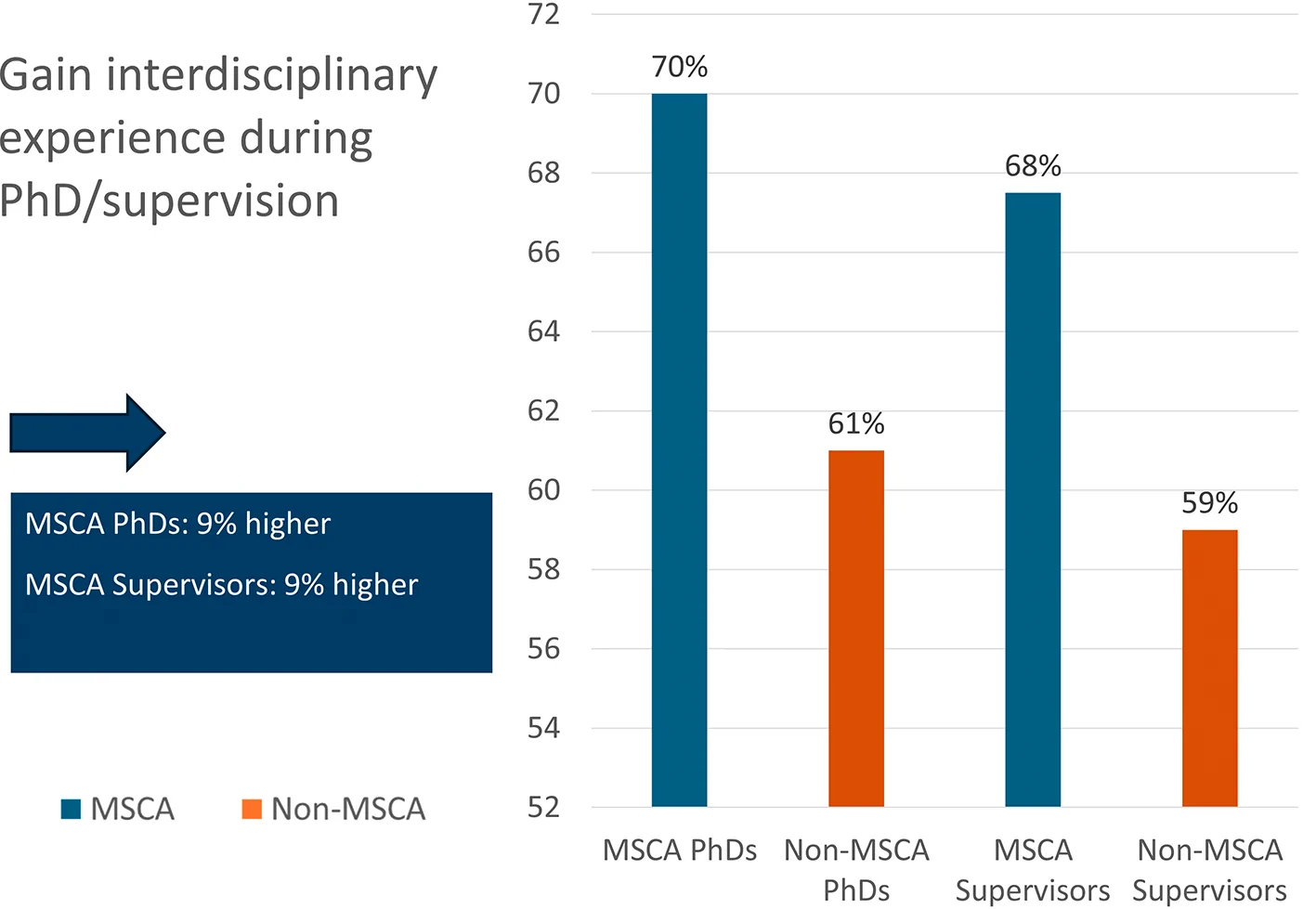
Gain interdisciplinary experience during PhD/supervision.

*4.1.2 Positive Impacts of the Triple “I” Mobility*

The transformative impact of the triple “i” mobility (international, intersectoral, and interdisciplinary) is strongly felt across various domains by MSCA fellows, surpassing that observed in their non-MSCA counterparts in numerous fields. MSCA participants report that their international experiences have significantly boosted their industry exposure, economic outcomes, environmental initiatives, sustainability, innovation and entrepreneurship efforts, and intersectoral collaborations ( Table 1). This broad influence underscores the strategic value of mobility at the heart of the MSCA framework, supporting EU and European Research Area objectives to promote multifaceted research, scientific excellence, a mobile research community, and a responsible future.

**Table 1.** Higher positive impacts of the triple “i” mobility experienced by MSCA PhDs and supervisors.

Higher positive impacts of the triple “i” mobility experienced by *MSCA PhDs*						
	International		Intersectoral		Interdisciplinary	
	MSCA PhDs	Compared to Non-MSCA	MSCA PhDs	Compared to Non-MSCA	MSCA PhDs	Compared to Non-MSCA
Industry exposure	69%	*25% higher*	85%	*19% higher*	75%	*29% higher*
Economic impact	66%	*19% higher*			73%	*13% higher*
Environmental impact	60%	*15% higher*			70%	*14% higher*
Sustainability	62%	*14% higher*			72%	*14% higher*
Innovation/entrepreneurship	73%	*10% higher*	77%	*13% higher*		
Change of country			75%	*17% higher*	85%	*10% higher*
Change of discipline			75%	*15% higher*	90%	*10% higher*
Intersectoral collaborations	92%	*10% higher*	N/A	*N/A*		
IPR strategy			70%	*27% higher*		
Knowledge of the market			83%	*10% higher*		
*Empty cells above indicate that the difference between MSCA and non-MSCA is below 10%*						

Higher positive impacts of the triple “i” mobility experienced by *MSCA Supervisors*						
	International		Intersectoral		Interdisciplinary	
	MSCA Supervisors	Compared to Non-MSCA	MSCA Supervisors	Compared to Non-MSCA	MSCA Supervisors	Compared to Non-MSCA
Industry experience	69%	*21% higher*	*81%*	*6% higher*	*69%*	10% higher
Intersectoral collaborations	91%	*8% higher*	N/A	N/A	95%	*6% higher*

Moreover, the intersectoral mobility of MSCA fellows has yielded remarkable positive effects in enhancing industry experience, fostering innovative and entrepreneurial ventures, facilitating country and discipline transitions, shaping intellectual property rights strategies, and enriching market insights ( Table 1). Higher positive impact on industy experience and scope for intersectoral collaborations is felt by MSCA supervisors as well ( Table 1). This emphasises the pivotal role of crossing sector boundaries in cultivating a versatile and agile research workforce.

Interdisciplinary mobility further amplifies these benefits, resulting in greater industry exposure, economic impact, environmental responsibility, sustainability efforts, and facilitating geographic and disciplinary mobility ( Table 1). Such mobility exemplifies the interconnected and synergistic nature of the triple “i” features of the MSCA, creating a powerful nexus of structured training and research environment that not only strengthens individual competencies but also catalyses systemic progress across research and professional landscapes.

Intriguingly, international mobility enhances intersectoral collaborations, while intersectoral and interdisciplinary mobilities facilitate greater transitions across countries and disciplines. Clearly, these dimensions are closely integrated, collectively enhancing the capacity and reach of MSCA fellows.

*4.1.3 Challenges to the Triple “I” Mobility*

MSCA supervisors encounter fewer challenges across various dimensions of international, intersectoral, and interdisciplinary mobility. As Table 2 illustrates, the obstacles faced by MSCA supervisors are notably less compared to their non-MSCA counterparts, facilitating smoother transitions and collaborations across borders and sectors. This advantage highlights the efficacy of the MSCA framework in empowering supervisors to competently manage complex mobility scenarios, enhancing collaborative potential within the research community.

**Table 2.** Significantly less challenges faced by MSCA supervisors.

Significantly *less challenges* faced by *MSCA supervisors*						
	International		Intersectoral		Interdisciplinary	
	MSCA Supervisors	Compared to Non-MSCA	MSCA Supervisors	Compared to Non-MSCA	MSCA Supervisors	Compared to Non-MSCA
Supervision support			68%	*10% less*	65%	*11% less*
Resource availability/funding challenges			72%	*12% less*	69%	*11% less*
Personal adaptation	60%	*16% less*	65%	*12% less*	64%	*16% less*
Academic isolation/integration (limited access to academic resources and support)	35%	*19% less*	40%	*16% less*	39%	*10% less*
Difficulty translating academic skills to industry-specific needs					47%	*9% less*
Publication difficulties			47%	*15% less*		
Navigating intellectual property rights and confidentiality agreements			63%	*14% less*		
Time management	39%	*16% less*				
*Empty cells mean the difference between MSCA and non-MSCA is below 10%*						

Similarly, MSCA PhD fellows demonstrate remarkable adaptability in navigating the triple “i” mobility, facing considerably fewer challenges in most concerned fields ( Table 3). Their experiences reflect significantly lower obstacles concerning resource availability, funding, and academic isolation. Nevertheless, they encounter slightly more difficulties in managing intellectual property rights and confidentiality agreements. Additional challenges, as identified through open survey responses, include visa issues, administrative and bureaucratic hurdles, intercultural differences, and communication barriers. These insights emphasise the importance of building upon current policy measures, enhancing support structures to ensure MSCA participants can excel in diverse research contexts while effectively navigating all possible challenges.

**Table 3.** Challenges faced by the MSCA PhDs compared to non-MSCA: significant differences.

Challenges faced by the MSCA PhDs compared to non-MSCA: significant differences						
	International		Intersectoral		Interdisciplinary	
	MSCA PhDs	Compared to Non-MSCA	MSCA PhDs	Compared to Non-MSCA	MSCA PhDs	Compared to Non-MSCA
Navigating intellectual property rights and confidentiality agreements	34%	*13% higher*			37%	*8% higher*
Time management	72%	*12% higher*				
Resource availability/funding challenges	43%	*17% less*	43%	*19% less*	46%	*19% less*
Academic isolation/integration (limited access to academic resources and support)					55%	*8% less*
Differences in work culture and expectations			71%	*8% less*		
Personal adaptation	75%	*8% less*				
*Empty cells mean the difference between MSCA and non-MSCA is below 8%*						

### 4.2 Supervision

The survey reveals a substantive shift in doctoral supervision models, with MSCA ITN/DN fellows 20% more likely to work within supervisory teams rather than under single supervisors ( Figure 7). This finding validates the MSCA supervision guidelines’ strategic emphasis on collaborative mentorship structures. Beyond improving research outcomes, team-based supervision also catalyses cross-institutional collaboration among principal investigators and fosters genuinely interdisciplinary training environments where fellows benefit from diverse epistemological frameworks and methodological expertise of multiple supervisors.

**Figure 7.**
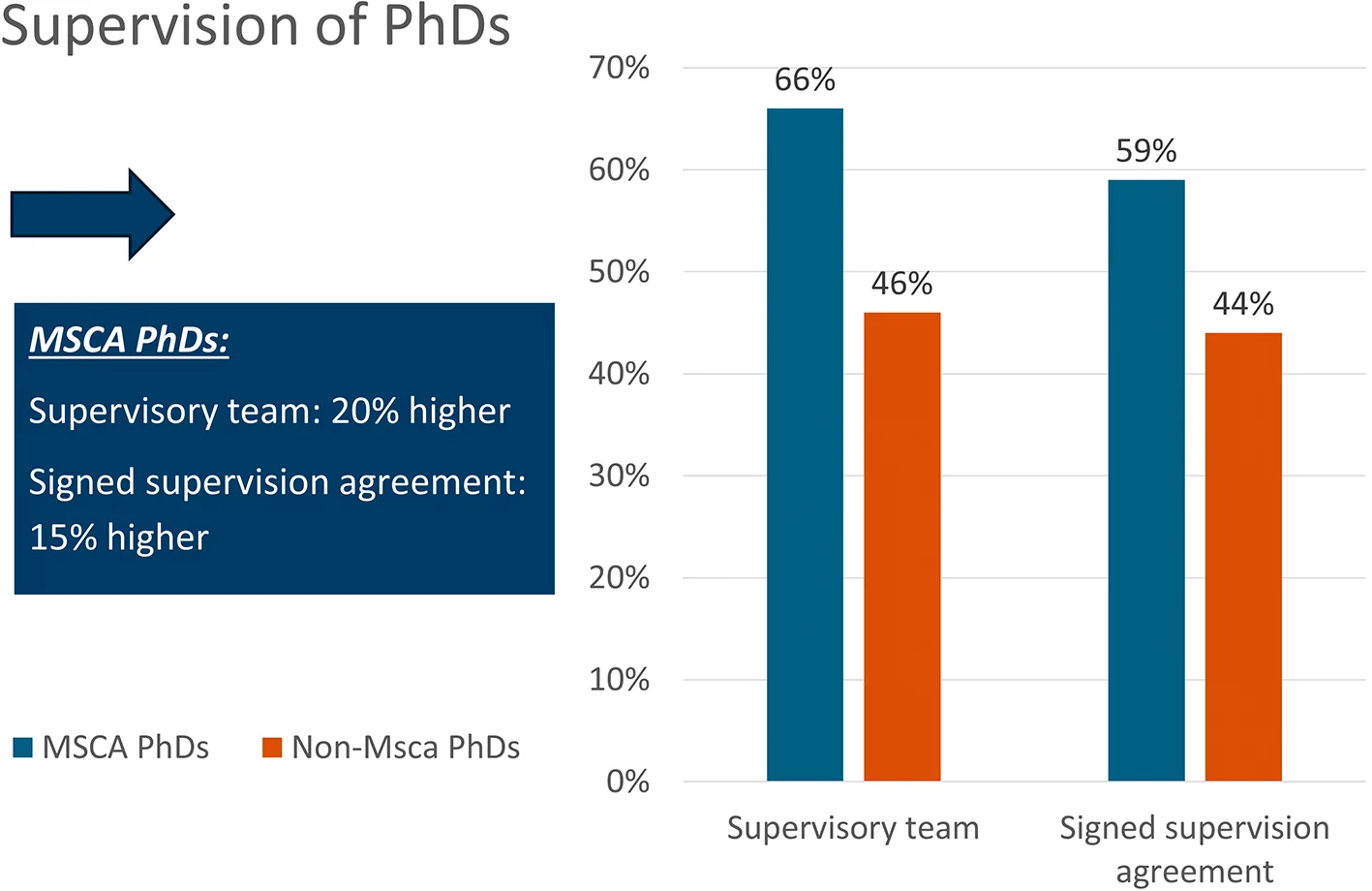
Supervision.

The formalisation of supervision arrangements represents another critical dimension of the MSCA framework. Survey data indicates that 15% more MSCA fellows undertake their research under formally concluded supervision agreements compared to their non-MSCA counterparts ( Figure 7). This procedural rigour in adherence to the MSCA Innovative Doctoral Training principles translates into measurable quality outcomes. While historical assessments showed 72% of ITN fellows rating their supervision positively in 2017, ^28^ the present study showcases an upward trajectory to 77% satisfaction among ITN/DN fellows, suggesting the MSCA’s sustained commitment to supervisory excellence. The revised MSCA Supervision Guidelines ^39^ are expected to reinforce this trend by emphasising the role of a broader supervision ecosystem that integrates fellows, supervisors, and institutions. Qualitative evidence from interviews underscores a distinctive pedagogical philosophy embedded within the MSCA DNs, i.e., the cultivation of researcher autonomy, which is prominent in MSCA supervisory practices. As many MSCA supervisors articulated in the survey, the programme systematically guides fellows to become future research leaders while instilling principles of research integrity, responsibility, and independent and critical thinking. This is in line with the general trend observed by Marie Curie Alumni Association (MCAA) across all MSCA actions that 80% of MSCA participants feel that their supervisors involve them in goal-setting and decision-making activities. ^27^ This approach reflects an emphasis on developing not merely technically skilled researchers but intellectually independent scholars capable of shaping their fields, and society at large going forward.

### 4.3 Training

“The MSCA [ITN/DN] programme creates a group of well-prepared scientists with exceptional skills and a well-developed network of relevant contacts. This makes them strong candidates to be the next generation leaders in their fields.” – MSCA Supervisor (Survey).

The MSCA framework promotes a structural mechanism for building multidimensional competencies. Survey findings demonstrate that compared to the non-MSCA, MSCA fellows yield demonstrable returns in transferable skills acquisition, reporting significantly enhanced social, networking, technical, and intercultural capabilities ( Figure 8). These competencies emerge from the structured, network-wide training architecture enhanced by triple “i” mobility that characterises MSCA programmes, encompassing cutting-edge disciplinary knowledge, original, and state-of-the-art trainings, international and intersectoral innovations, and promotion of adaptable professional skills responsive to evolving labour market demands.

**Figure 8.**
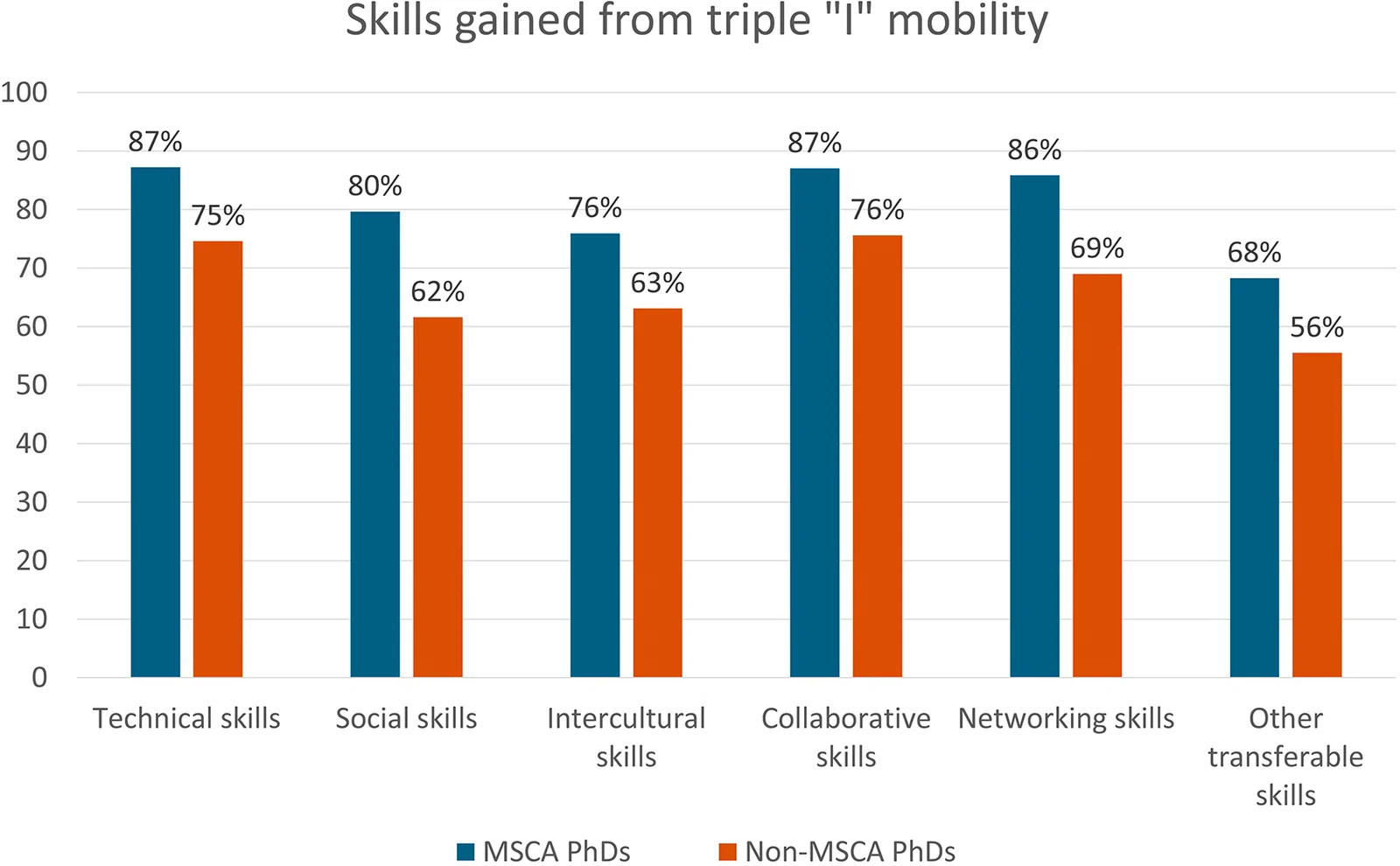
Skills gained from triple “i” mobility.

The MSCA initiatives like ITN and DN have been visionary in promoting excellent science through comprehensive training of the researchers. The MSCA ITN/DNs’ overarching excellence award criteria go beyond research skills, and augment doctoral training further through mandatory international and intersectoral exposure from mobility, career development plans signed by both the supervisors and the PhD fellows, and high quality transferable skills training. As one of the MSCA supervisors writes in response to the survey, the MSCA DN “provides a significant gain in skills that are transferable across sectors, preparing students comprehensively for varied career paths”.

For almost all types of trainings analysed in the survey, the MSCA fellows consistently expressed higher satisfaction with the training they received for developing several crucial skills for research and professional development ( Figure 9). The MSCA fellows expressed especially higher satisfaction than the non-MSCA in their training for networking, public speaking and presentation, teamwork, and ethical awareness. These transferable skill trainings remain vital for equipping researchers with skills such as adaptability, problem-solving skills, and innovative and entrepreneurial thinking which are necessary to succeed in diverse career paths. The rigorous training offered to the MSCA fellows not only enhances research capabilities of the fellows but also prepares them to tackle complex questions beyond academia. As one of the PhD fellows notes in the survey, their MSCA PhD experience has been enriching, especially because it included vital soft skills training and professional mentoring.

**Figure 9.**
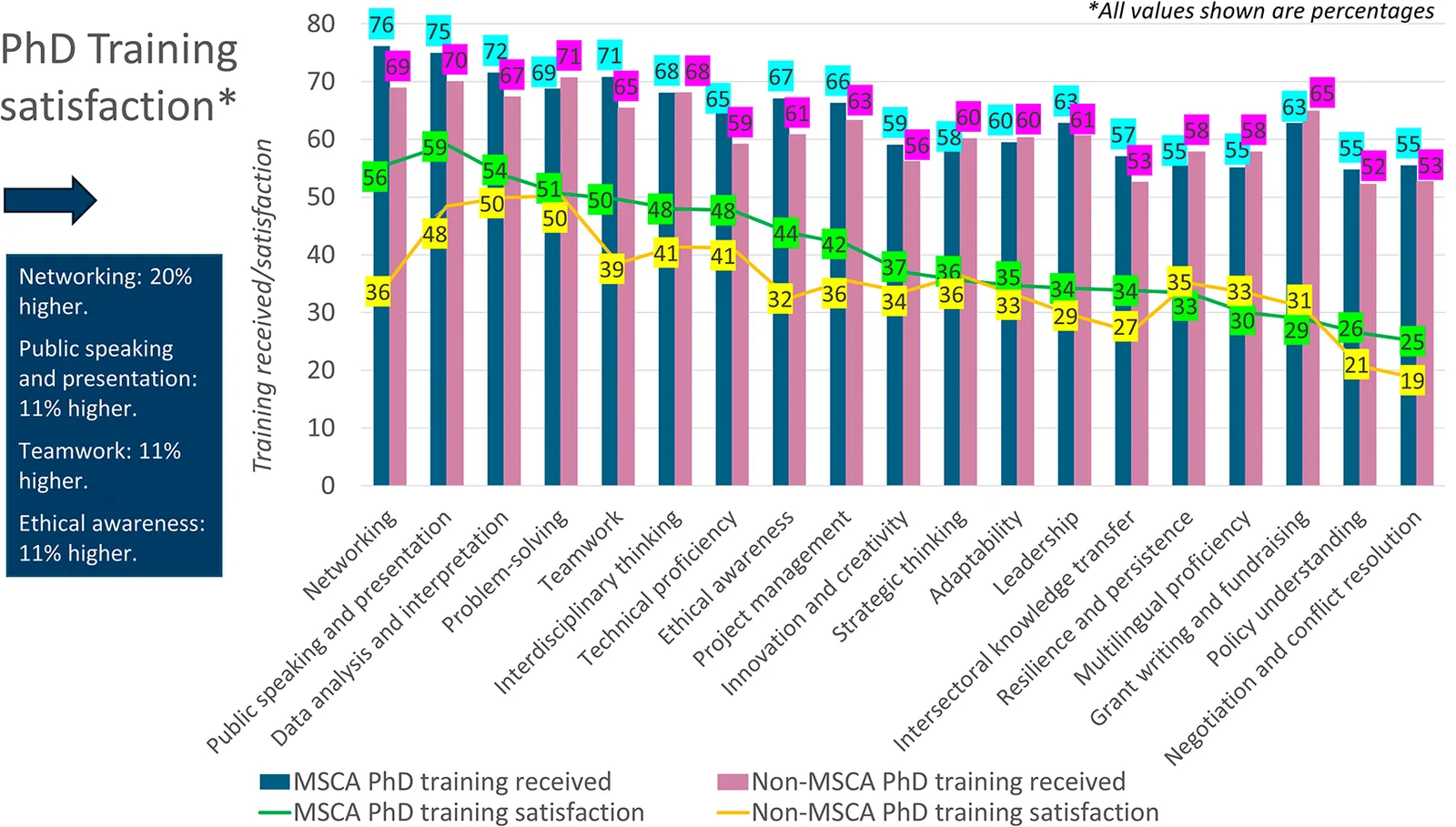
Training satisfaction.

### 4.4 Social Innovation and Working Environment

The survey demonstrates that the MSCA PhD researchers benefit from better working conditions compared to their non-MSCA counterpart. Under the MSCA, it is required that all PhD fellows are recruited with an employment contract and social security coverage (verified internally using the CORDA database), whereas only 43% and 77% of non-MSCA fellows report being recruited with an employment contract and social security respectively (data not shown).

There are notable differences in funding and employment conditions for PhD fellows across European countries. For example, while in some countries, fellows receive an employment contract and social security, in other countries, they may receive a fellowship. The MSCA ITN/DN programmes provide a framework for standardisation of these varying working conditions by emphasising the requirement of recruiting fellows under an employment contract or other (equivalent) direct contract, with full social security coverage, while also highlighting the importance of family-friendly working conditions, well-being, and access to social security rights and pensions. ^40, 41^

For the MSCA fellows, satisfaction with resources and financial remuneration has been generally high. ^12^ The present study reaffirmed this trend with its finding that a 33% higher number of MSCA fellows find their salary to be highly or very highly sufficient to manage costs of living, compared to their non-MSCA counterpart ( Figure 10).

**Figure 10.**
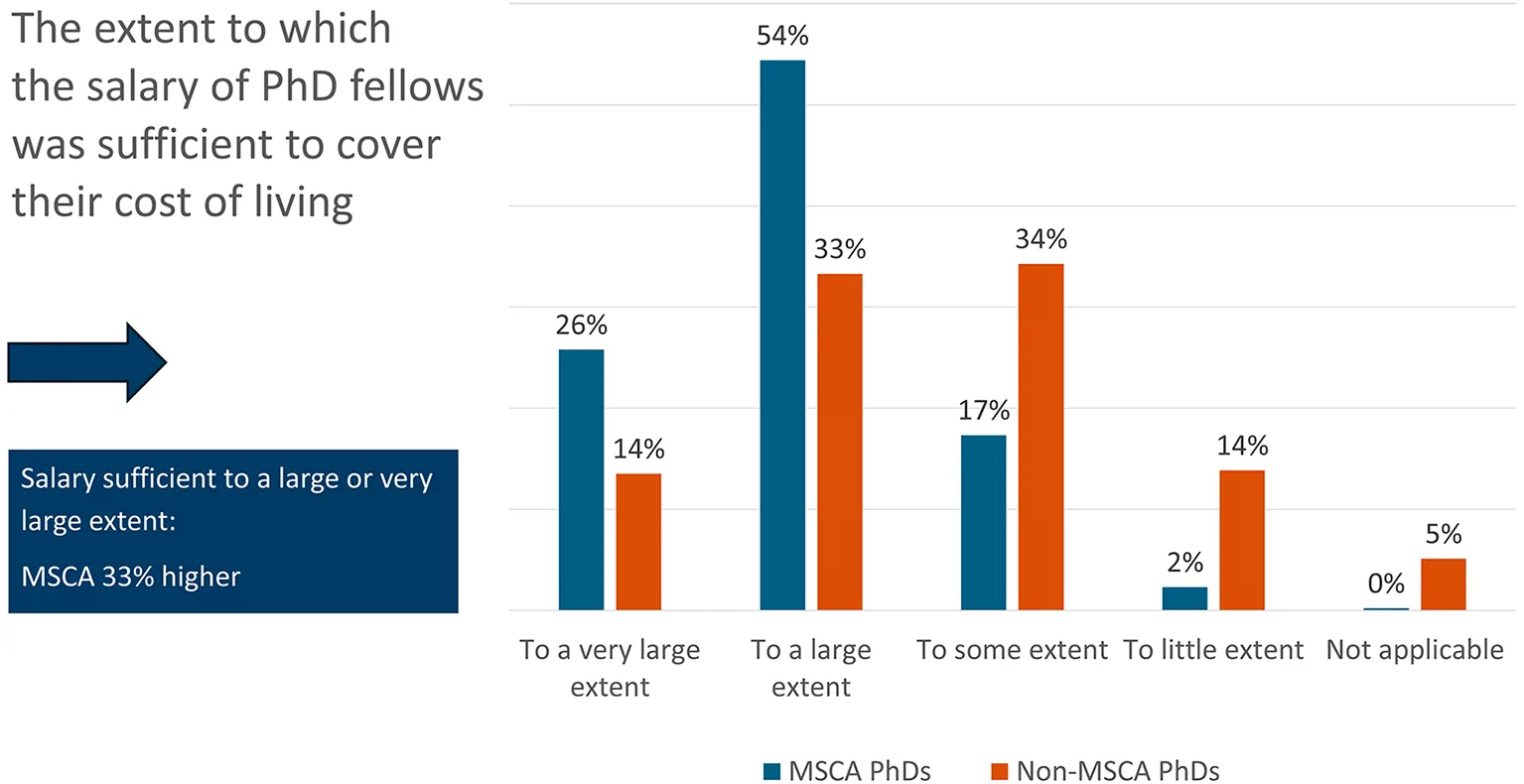
To what extent was the PhD fellows’ salary sufficient to cover cost of living?.

As one of the MSCA PhD fellows notes in the survey, “MSCA PhD funding helps one to concentrate fully on studies without worrying about financial issues, enabling deeper focus and innovation in research”. The MSCA supervisors also expressed general consensus on this. As one MSCA supervisor notes, “Training and networking opportunities combined with a competitive salary attract top-tier talent while ensuring sustained motivation” (source: survey).

Although a majority of the MSCA supervisors and PhD fellows indicated that the MSCA salary is highly sufficient in terms of cost of living, a 19% MSCA fellows and 8% MSCA supervisors indicated that the MSCA salary is sufficient only to some or little extent which warrants further investigation. The table ( Table 4) below lists the countries where such perceptions have been noted.

**Table 4.** MSCA participants reporting salary too low to meet costs of living (by country).

Percentage of MSCA participants (supervisors and fellows) who think the salary of MSCA fellows is not sufficient to meet the costs of living (by country)			
Country	Raw percentage ^*^	Adjusted percentage ^**^	Number of MSCA respondents
Norway	27.3%	23.5%	77
Switzerland	23.6%	21.7%	123
Bulgaria	75.0%	21.0%	4 ^***^
Turkey	44.4%	20.9%	9 ^***^
Portugal	20.9%	19.4%	110
Germany	19.5%	19.2%	560
Croatia	30.8%	18.9%	13 ^***^
Slovenia	24.0%	18.4%	25 ^***^
Netherlands	18.1%	17.8%	337
Ireland	18.7%	17.5%	91
Slovakia	21.4%	16.2%	14 ^***^
Iceland	50.0%	16.1%	2 ^***^
Sweden	16.3%	15.9%	166
Belgium	16.0%	15.8%	256
Luxembourg	22.2%	15.8%	9 ^***^
United States of America	33.3%	15.6%	3 ^***^
Estonia	14.3%	14.0%	14 ^***^
Serbia	14.3%	13.9%	7 ^***^
Finland	13.8%	13.8%	94
Latvia	12.5%	13.5%	8 ^***^
Israel	13.0%	13.5%	23 ^***^
Romania	13.0%	13.5%	23 ^***^
Poland	11.9%	12.7%	42
Greece	12.0%	12.5%	83
France	11.0%	11.2%	319
Italy	10.0%	10.2%	480
Cyprus	4.3%	9.7%	23 ^***^
United Kingdom	9.1%	9.4%	429
Spain	8.7%	9.0%	425
Denmark	7.8%	8.7%	167
Czechia	4.0%	7.7%	50
Austria	4.1%	5.8%	146

^*^ Global average of the sample = 13.8%.

^**^ K = 30 (Prior strength used for Bayesian smoothing. This value was chosen to stabilise estimates for low-sample groups while preserving observed variation in larger samples.)

^***^ Sample size not reliable.

The countries where some of the fellows feel that their salary may not be fully sufficient come from Belgium, Germany, Ireland, the Netherlands, Norway, Portugal, Sweden, and Switzerland (see “adjusted percentage” in Table 4). ^x^ The results generated from this study as shown in the table above mostly correspond with the trends observed by REA in the recent years which shows that the MSCA salary rates may not be as competitive anymore as they used to be in some countries that now offer similar or even higher salary levels demonstrating a structuring effect of the MSCA in doctoral salaries. Other reasons for the countries with higher salary in the local level compared to the MSCA may include increasing national focus on research and innovation, adjustment for inflation etc.

83% of MSCA supervisors noted that they would not have been able to conduct the research project without the MSCA funding (data not shown). The study also shows that 33% more MSCA supervisors find the budget for research, training and mobility sufficient to a large or very large extent in MSCA-funded doctoral projects compared to others ( Figure 11). It is reflected in one of the non-MSCA supervisors’ comments that “The funding level [in MSCA] is much higher than other PhD funding sources, allowing for innovative and ambitious research projects” (source: survey). Non-MSCA PhD fellows also recognise that “the funding from MSCA is substantial compared to other PhD options, which enables a richer research framework and global exposure” (source: survey).

**Figure 11.**
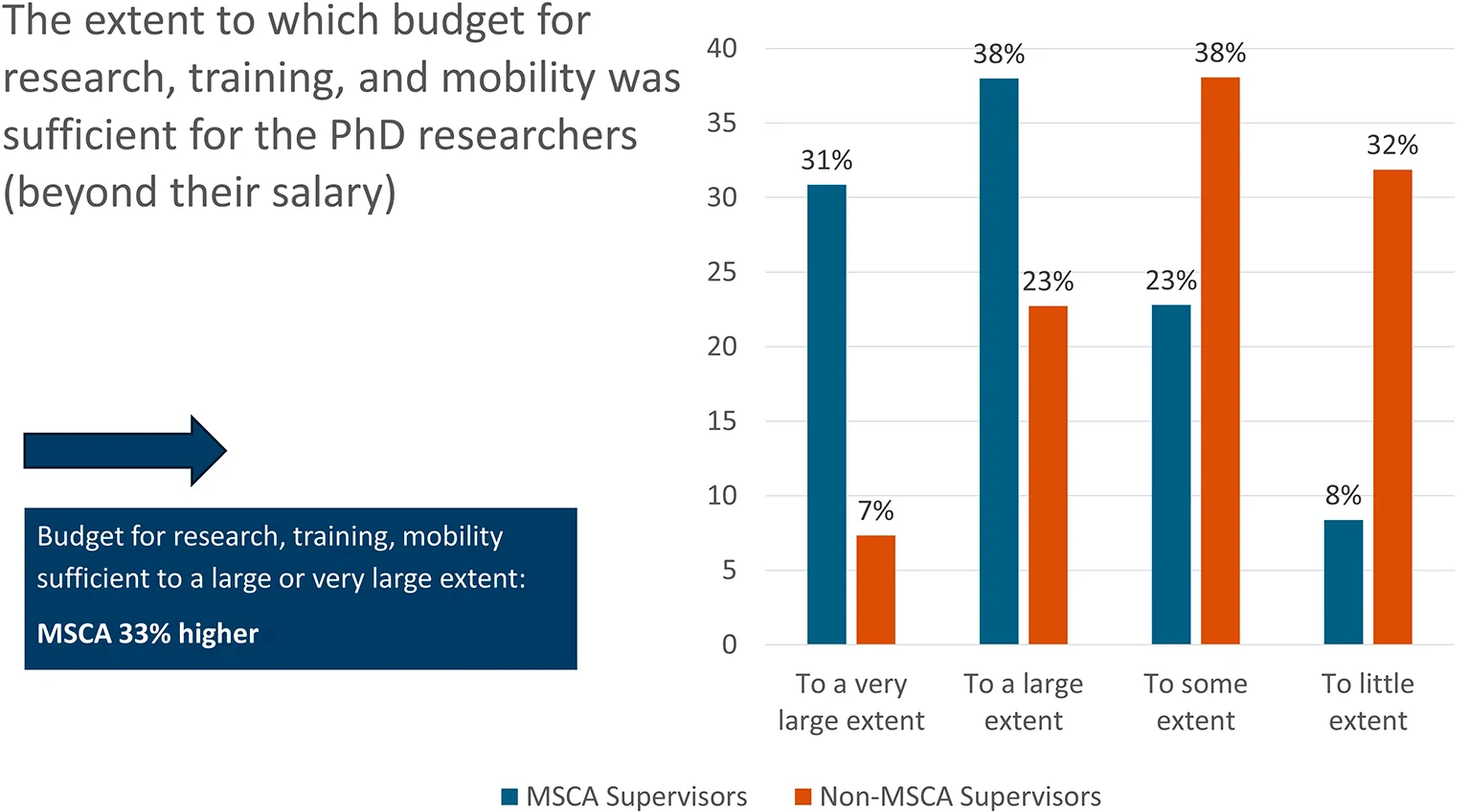
Budget for research, training, and mobility for the PhD researchers beyond their salary.

Amongst all the panels funded under the MSCA ITN and DN frameworks, Mathematics (MAT) is the panel in which the funds available for research, training and mobility are sufficient to the highest extent followed by Physics (PHY), Social Sciences and Humanities (SOC), Environment and Geosciences (ENV), Information Science and Engineering (ENG), and Chemistry (CHE) ( Figure 12). In the panels of Life Sciences (LIF), and Economic Sciences (ECO), the funds available for research, training and mobility could perhaps benefit from an increase ( Figure 12). ^xi^

**Figure 12.**
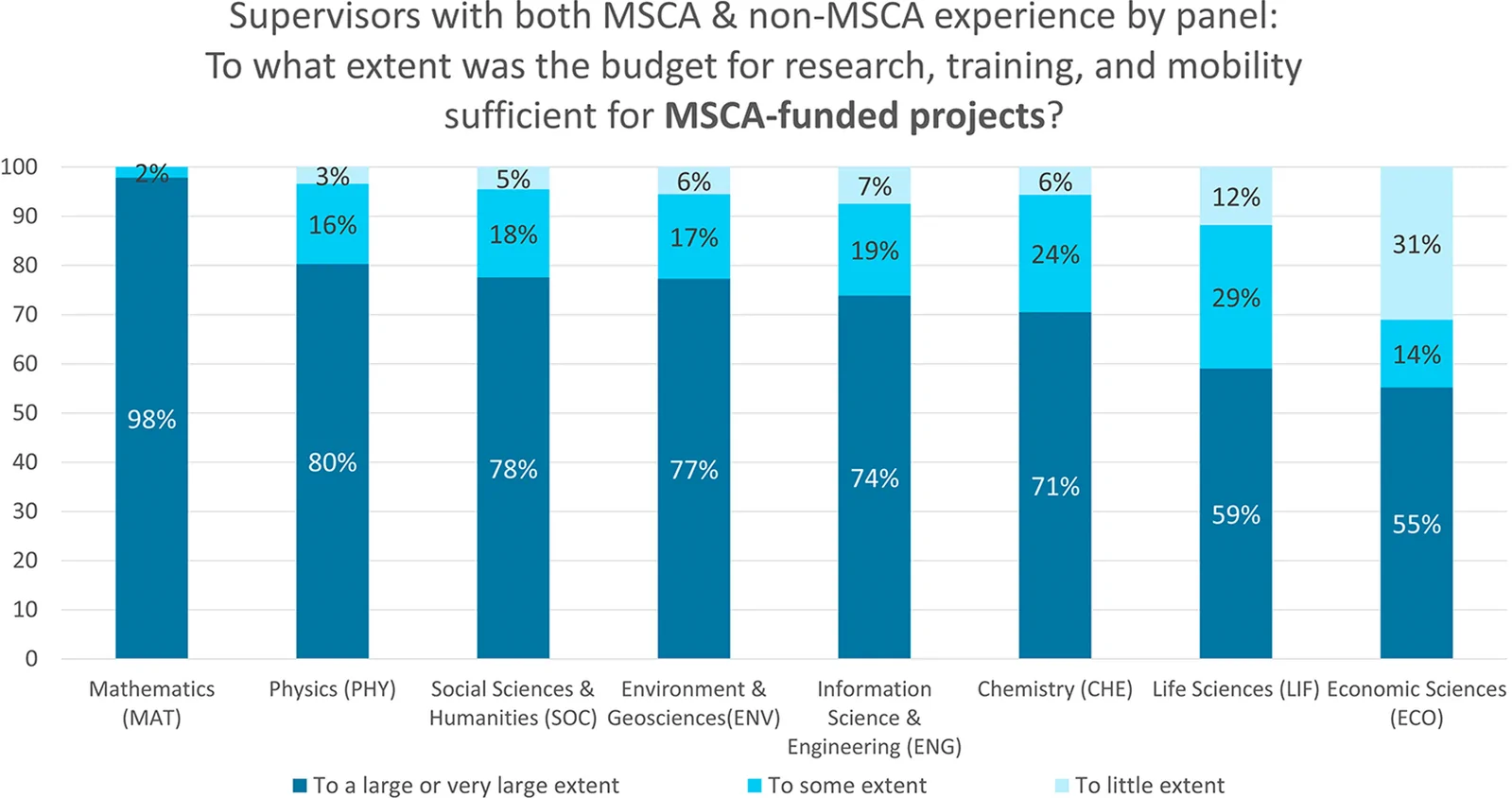
MSCA Supervisors’ opinion on budget for research, training, and for MSCA-funded projects by panel.

Although the overall funds available for research, training and mobility are higher in all panels in MSCA compared to the non-MSCA, the least number of supervisors felt that Mathematics (MAT), and Social Sciences and Humanities (SOC) received sufficient funding (to a large or very large extent) for these criteria under non-MSCA projects. More supervisors, however, expressed that Economic Sciences (ECO), Environmental and Geosciences (ENV), and Information Science and Engineering (ENG) receive relatively higher funding for research, training and mobility under non-MSCA (compare Figure 12 and Figure 13).

**Figure 13.**
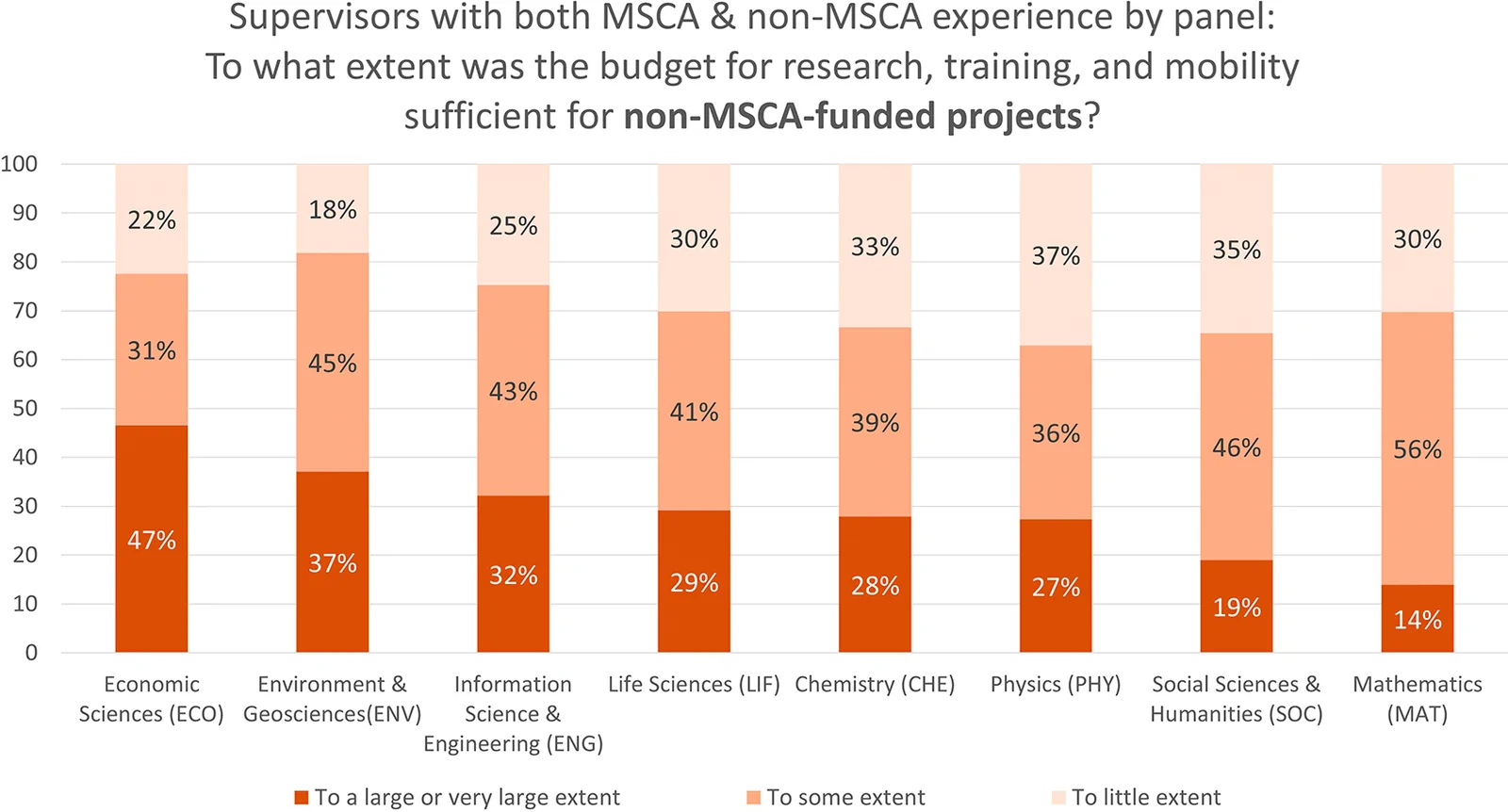
MSCA Supervisors’ opinion on budget for research, training, mobility for non-MSCA-funded projects by panel.

Although the MSCA DNs, like the previously concluded ITNs, provide three years of secure funding and excellent working conditions, some fellows are also challenged by a structural difference in their institution which may require four years of dedicated research for the completion of their PhD which is also the standard duration of PhD in several universities. 78% of the MSCA fellows (28% more than non-MSCA) reported that the funding period (three-years for MSCA) poses challenges in completing their PhD ( Figure 14).

**Figure 14.**
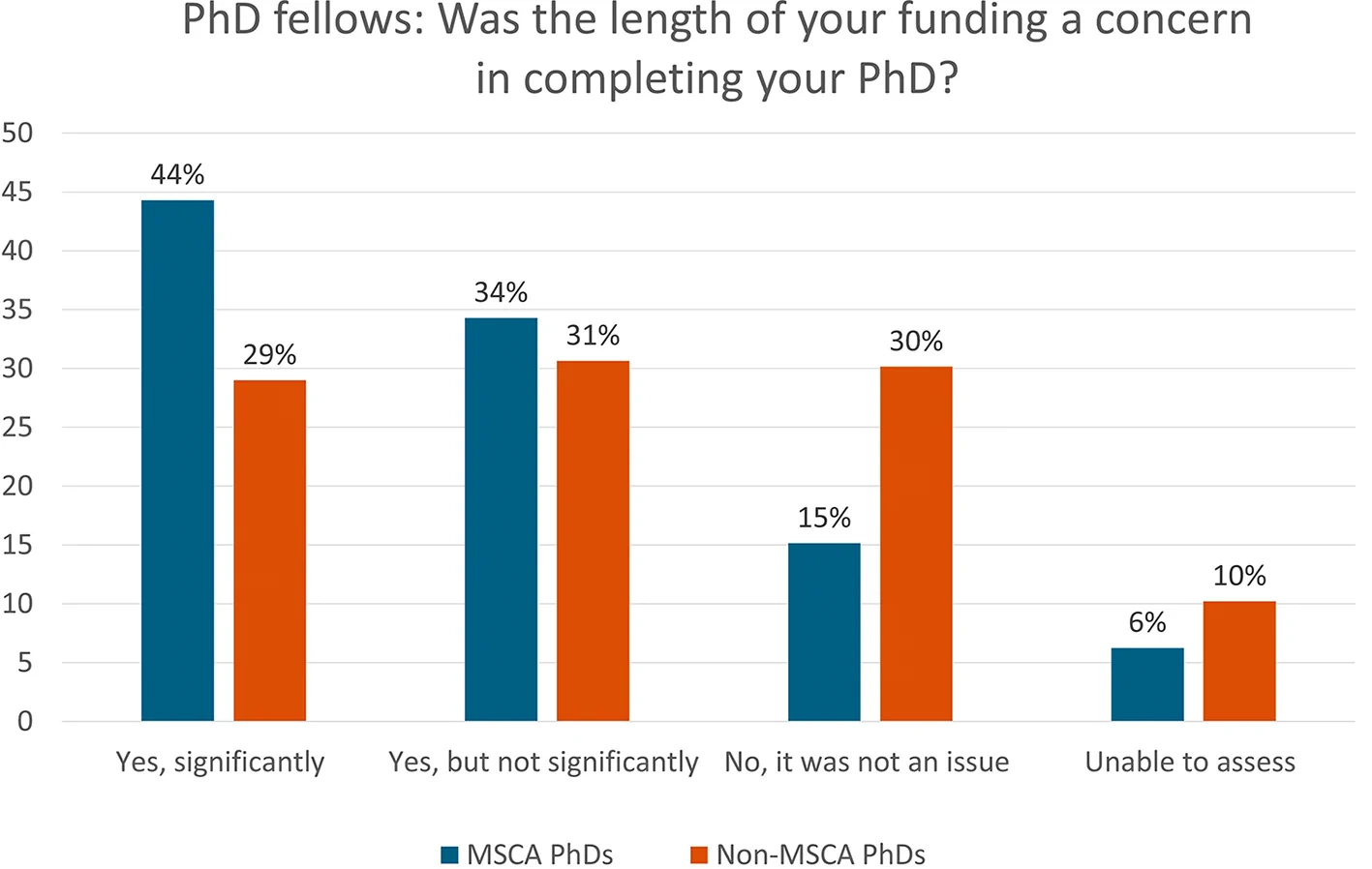
Length of funding as a concern in completing PhD.

This structural inconsistency also pushes the MSCA DN fellows to look for alternative funding sources during the final year of their PhD. Therefore, to enhance the doctoral experience of future DN fellows, increasing their funding duration from three to four years and the project duration from four to five years may be beneficial helping fellows maintain research continuity without needing to search for alternative funding in the final stages of their doctorate.

### 4.5 Career Perspectives and Networking Opportunities

“The ability to be mobile inside the network, to have these contacts at the very onset of the research career, I think that is an advantage that cannot be overestimated because it is indeed the best start to a researcher’s career and to have that network cover also interdisciplinary and intersectoral features […] I think that is quite unique. […] The added value of the programme is very high for the organisations, the PhD candidates and the network as such”. *–* Stakeholder of doctoral research and training in Europe (Interview).

The survey reveals that the MSCA programme has had a clearly positive impact on the employability of its graduates. A 12% higher number of the MSCA supervisors (60%; compared to 48% non-MSCA) have observed in the survey that the employability of PhD fellows has increased after their PhD in the last decade ( Figure 15). As one of the MSCA supervisors sums up many others’ voices aptly:

**Figure 15.**
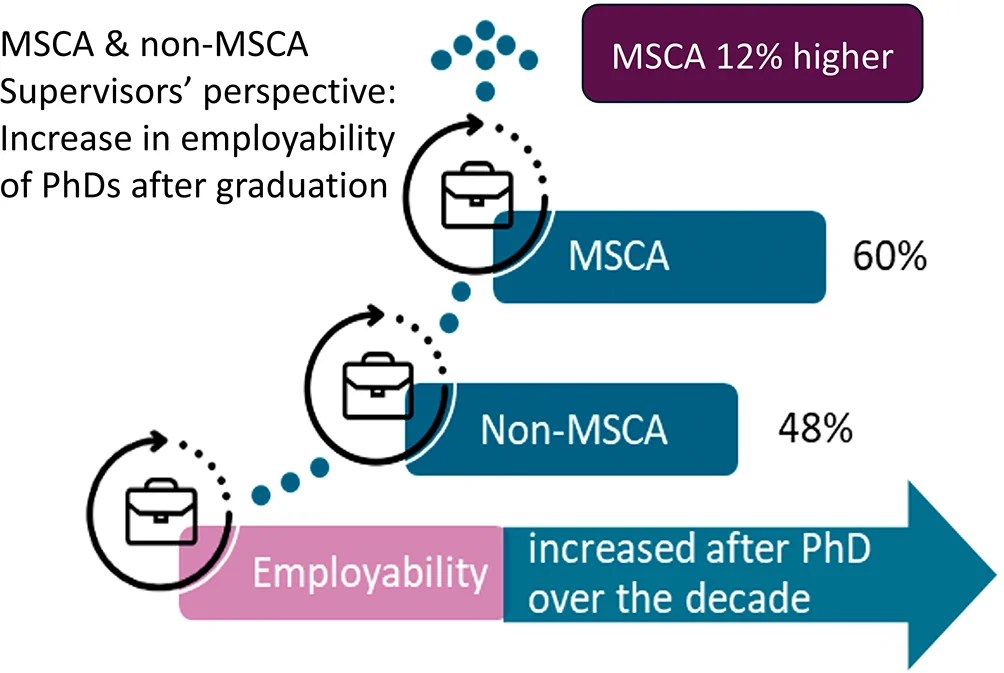
Supervisors’ opinion on increase in employability of PhDs after graduation.

“It [MSCA ITN/DN] is one of the most prestigious PhD programmes in Europe, with outstanding support in professional skills development and career readiness”. – MSCA Supervisor (Interview).

The fellows’ responses match with this trend observed by the supervisors above. Compared to the non-MSCA, 12% more MSCA PhD graduates (92%) secure employment within one year of graduation, demonstrating higher employability ( Figure 16). This underscores the MSCA ITN and DN programmes’ effectiveness in improving career prospects through comprehensive training, including international, intersectoral, interdisciplinary, and transferable skill development *.*

**Figure 16.**
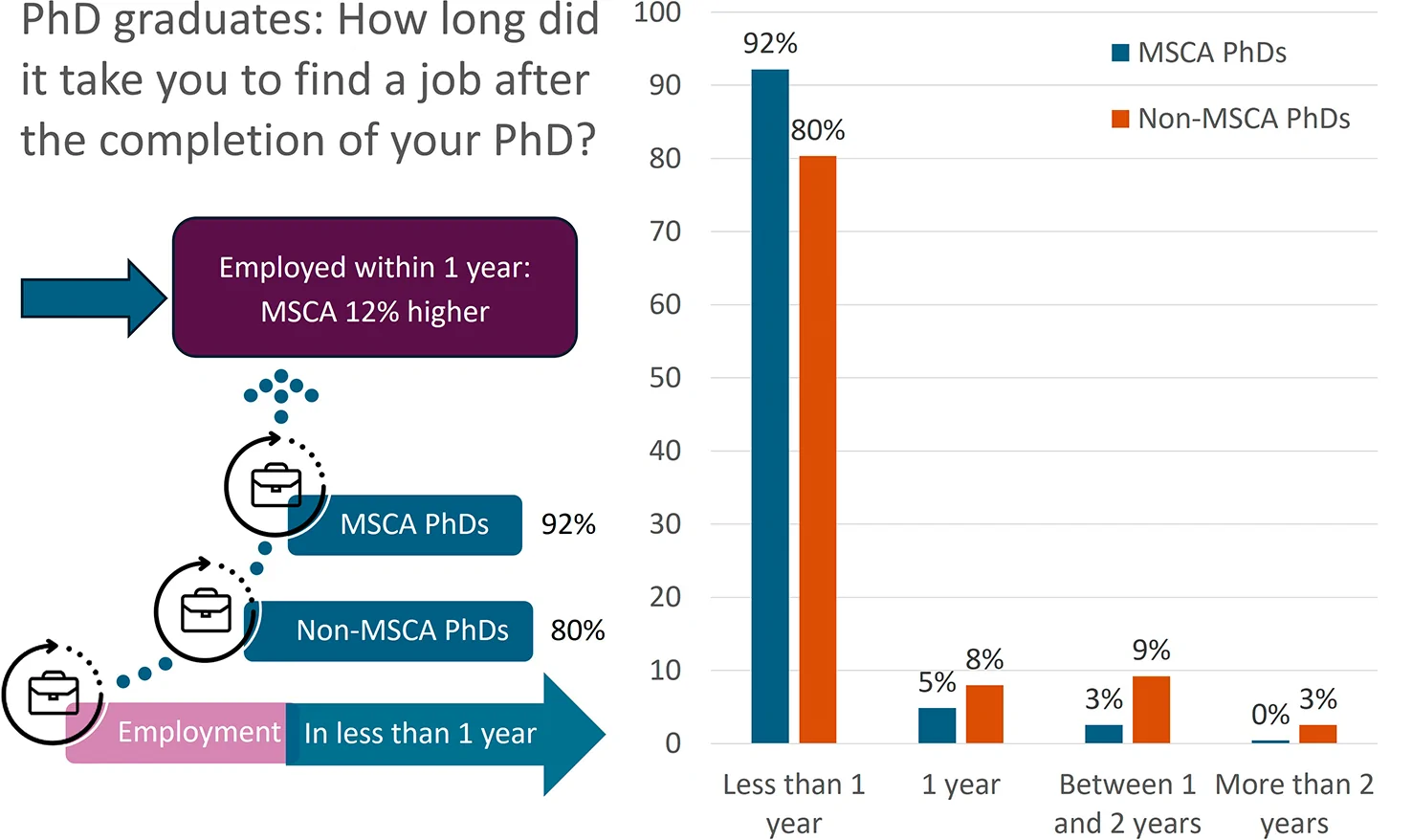
How long did it take you to find a job after the completion of your PhD?.

While only 15% of the non-MSCA fellows pursue a job outside the field of their core training after PhD, 8% more MSCA fellows venture into new fields that match their core training only to some or no extent ( Figure 17). A move into a new field requires higher intersectoral exposure, and relevant skills; if not field-specific skills, then at least transferable skills. The fact that a higher number of MSCA fellows find employment in new fields with little connection to their core field of research training is an implicit demonstration of the transformative role that mobility, diverse exposure, and transferable skills development play in the career building of the MSCA fellows.

**Figure 17.**
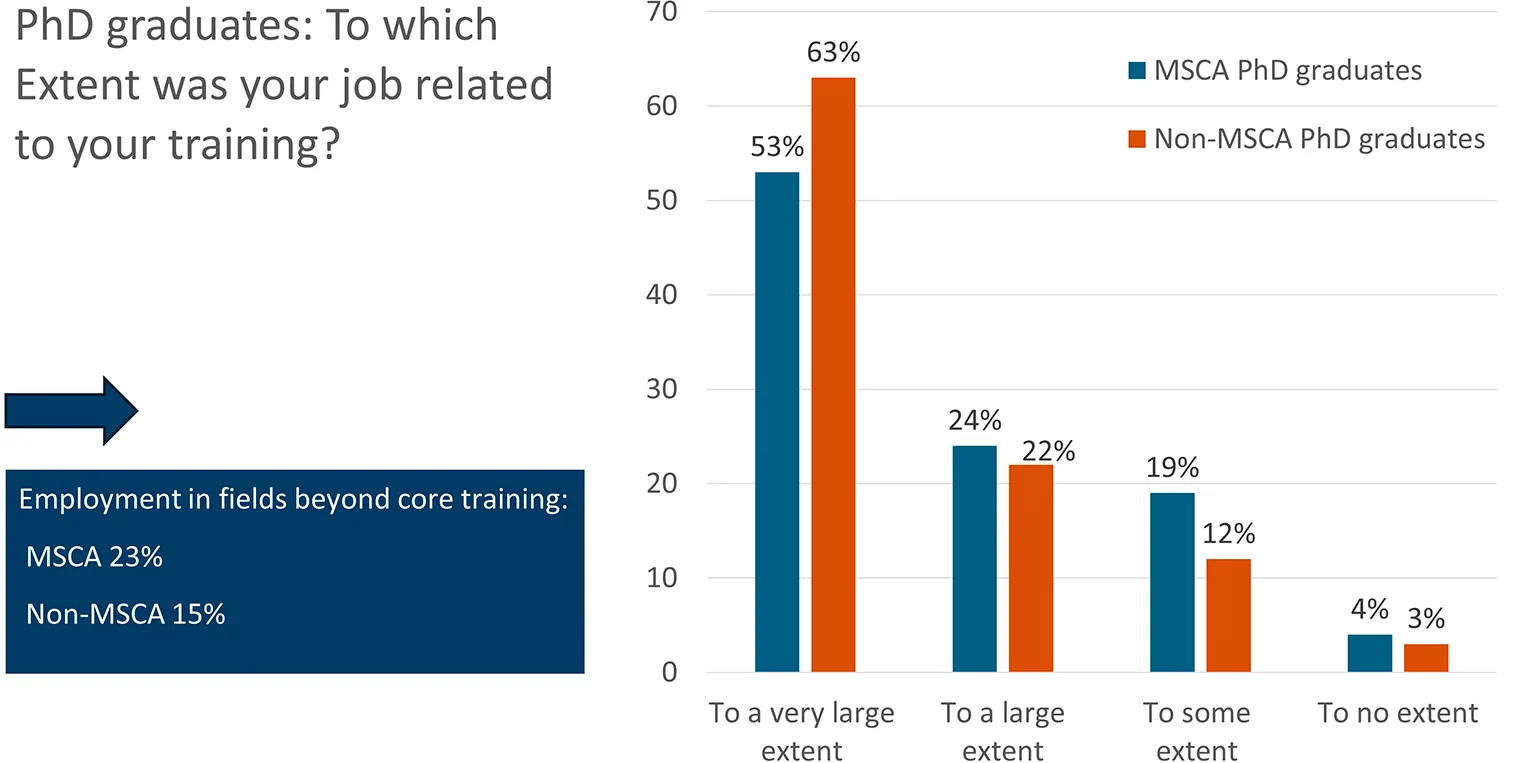
To what extent was your job related to your training?.

Compared to non-MSCA (62%), 9% more MSCA supervisors (71%) have observed that networking in the scientific community has been positively fostered in the past decade. As the findings discussed in the previous sections suggest, networking opportunities in the ITN and DN have been one of the major attractions of the programme which has been fostered especially by the MSCA’s emphasis on international, intersectoral and interdisciplinary mobility.

The results of the survey demonstrate that triple “i” mobility has significantly enhanced the employability and career opportunities of the MSCA fellows ( Figure 18). The highest difference between the MSCA and the non-MSCA in this field is observed in relation to intersectoral mobility. A 12% higher number of MSCA fellows expressed that intersectoral training enhanced their employability and career opportunities. The fact that a higher number of MSCA fellows found intersectoral mobility enhancing their employability and career prospects substantiates the trend that more MSCA fellows find it easier to move to fields outside their core training during PhD. It can be observed further in the trends of the sector employment of PhD fellows after graduation. As shown earlier, over three times more MSCA fellows find employment in non-academic sector after graduation compared to non-MSCA PhDs ( Figure 5).

**Figure 18.**
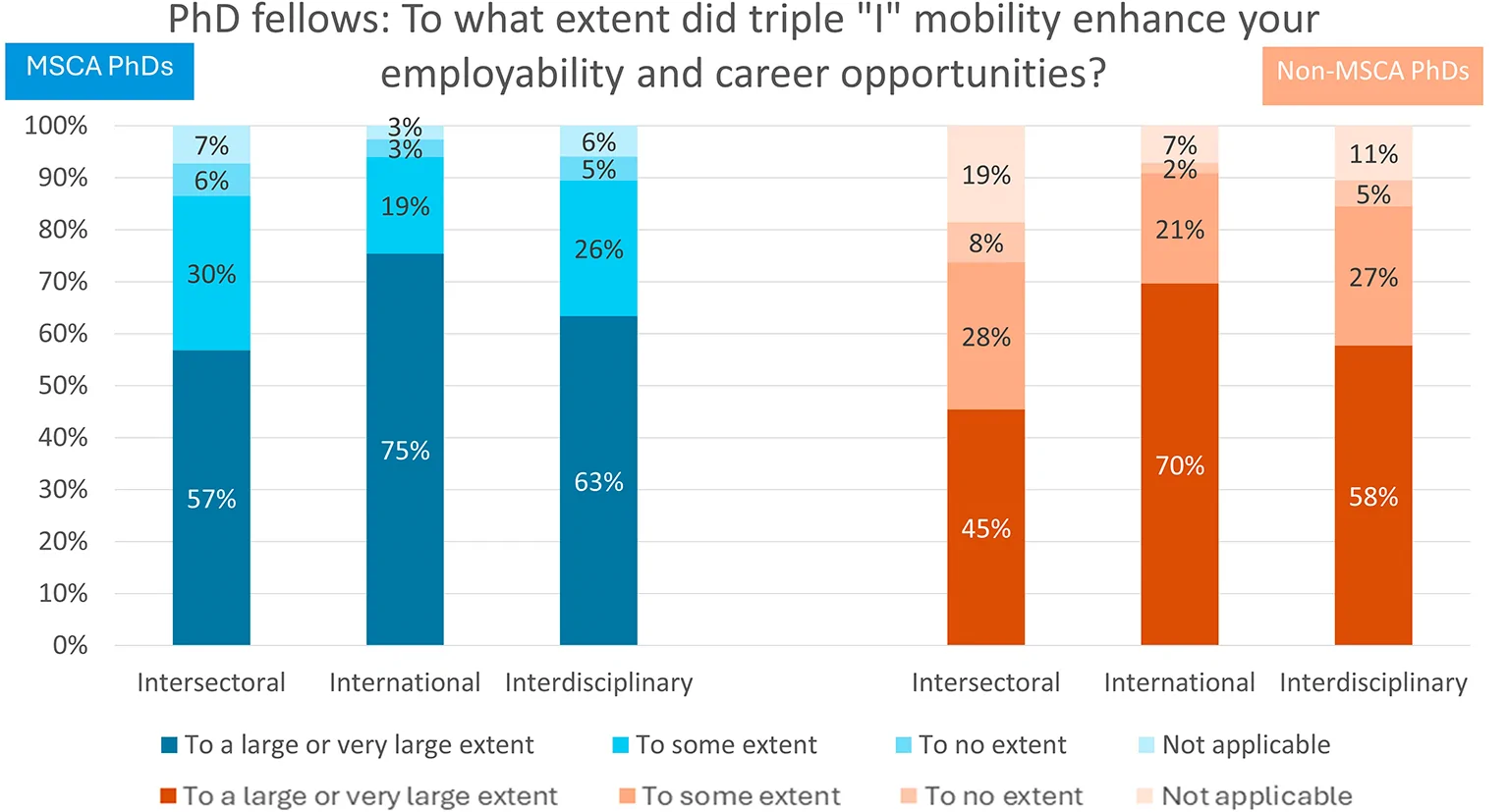
To what extent did triple “i” mobility enhance employability and career opportunities?.

### 4.6 MSCA Structuring Effect

“[The MSCA programme] pushes the creation of common frameworks among institutions, advancing regional collaborations and research standards.” – Non-MSCA supervisor (Survey).

By promoting standardisation and harmonisation ^xii^ of higher education, the MSCA have left a deep structuring effect across institutions which has been felt by a majority of the supervisors who participated in the survey ( Figure 19). Although it might be difficult for individual researchers to measure structuring effects at institutional levels, a majority of both MSCA and non-MSCA supervisors agree that harmonisation and standardisation across institutions have increased in the past decade and have had an essentially positive impact on doctoral programmes ( Figure 19). The main positive changes observed by the supervisors include enhanced skills development for researchers, and better mentoring and career guidance (data not shown). Upon being asked what factors influenced positive structuring effects in doctoral programmes, a majority of the supervisors indicated that it is the promotion of best practices and benchmarking, whereas the MSCA supervisors specifically attributed these positive changes to their access to the MSCA doctoral network framework (data not shown).

**Figure 19.**
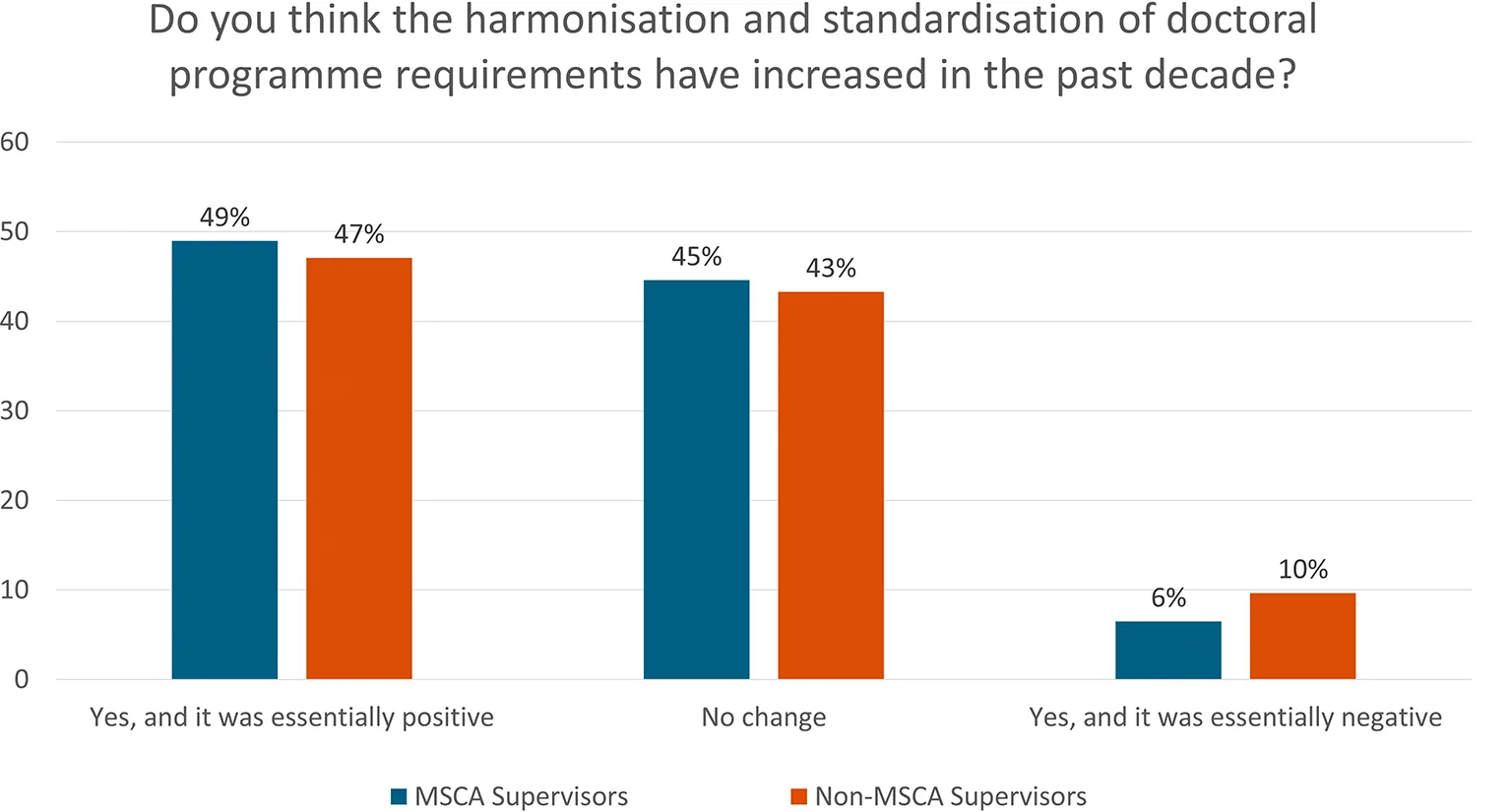
Supervisors’ opinion on harmonisation and standardisation of doctoral programme requirements in the past decade.

The MSCA DNs, following the legacy of the ITNs, contribute structurally by improving doctoral education systems across Europe which reflects in its prestige and recognition. The European Charter for Researchers, embedded in the MSCA not only structure the MSCA-funded PhD programmes but also leave a strong impact on the operation of all doctoral programmes at pan-institutional levels. Over time, the emergence of thousands of collaborations facilitated by the MSCA has structured the European research landscape, particularly fostering international as well as intersectoral collaborations, ^34^ leading to research excellence results and tangible innovation outputs. ^7^ As one of the supervisors interviewed acknowledges:

“The MSCA ITN/DN has a significant impact on the scientific community because the workshops, schools, and conferences organised by the MSCA Doctoral Networks attract a bigger researcher community. They bring people together, providing a fertile ground for follow-up research activities based on the network”. – MSCA Supervisor (Interview).

This offers a compelling illustration of the structuring effects of the MSCA ITN and DN, which help shape the research environment in ways that extend well beyond the immediate consortium members.

The MSCA DNs, like the ITNs, operate at two complementary levels: fostering collaboration between individual researchers while simultaneously building sustainable inter-organisational partnerships that extend beyond individual project lifecycles. The programmes’ strategic emphasis on bridging academic institutions with SMEs and non-academic organisations creates a structuring effect, enhancing researchers’ innovation potential across sectors while strengthening Europe’s broader research and innovation ecosystem. ^7^

The structuring effect of MSCA ITN and DN is also closely connected to the bottom-up approach that characterises MSCA funding. In contrast to many national PhD programmes, MSCA ITNs and DNs promote interdisciplinary research through their strong emphasis on a bottom-up approach to research funding and the integration of multiple disciplines. This commitment to cross-disciplinary collaboration not only expands the scope of research but also stimulates innovative solutions, ensuring that MSCA graduates are well prepared to address complex, multifaceted challenges within an increasingly dynamic scientific and professional landscape. Consequently, the bottom-up nature of the programme was identified as a key factor in fostering scientific excellence and advancement.

Many respondents commented on the bottom-up approach of the programme as a distinctive strength, recognizing its structuring effects in fostering researcher-driven, curiosity-led collaboration. By avoiding prescriptive thematic priorities, it shapes doctoral education and research through researcher-driven choices, fostering diverse international networks, training models, and research domains rather than concentrating funding in a few policy-driven areas. As one participant remarked in the survey, the programme’s aspects of “bottom-up approach, network, and substantial funding make it possible to set up really ambitious and impactful projects.” The bottom-up nature of the MSCA ITN/DNs makes the programme substantially democratic and open-ended, fostering novelty in interdisciplinary dialogue. The bottom-up mechanism, thus, enhances scientific autonomy while democratizing access to collaborative networks and structuring sustainable collaborations. The programme also facilitates future bottom-up collaborations amongst early career researchers by setting examples of good practices in bottom-up research. Institutions build networks and training programmes that reflect their local strengths, which can persist beyond the funded project. This creates an alignment of programmes with institutional strategy and regional ecosystems (e.g., industry partnerships, interdisciplinary initiatives), which in turn creates long-lasting European networks, which can have a structuring effect. As another participant notes:

“The network aspect of the programme in which network-wide training is given to PhD fellows at the same stage is indeed an added value. This is both efficient and introduces the PhD fellows to like-minded peers with whom ‘bottom-up’ collaborations can be established.” – MSCA Supervisor (Survey).

The programme’s bottom-up design, therefore, acts as a catalyst for sustaining future collaborations. Its structuring effects benefit not only established researchers but also encourage early-career researchers to envision international, intersectoral, and interdisciplinary collaborations from the outset of their doctoral training, enabling transformative research.

The networks formed during the project persist beyond the projects’ duration, often continuing as collaborations that influence doctoral education standards and mobility cultures in the European Research Area. Partnerships often spread best practices (e.g., supervision models, industry secondments), contributing to a more harmonised doctoral landscape without a centralised mandate. This network effect is a key mechanism through which MSCA DN delivers structural impact at the European level.

### 4.7 Strengths of the MSCA Compared to non-MSCA

According to the findings of the study, a vast majority (94%) of the participants agree that the MSCA ITN and DN funding have several added values ( Figure 20).

**Figure 20.**
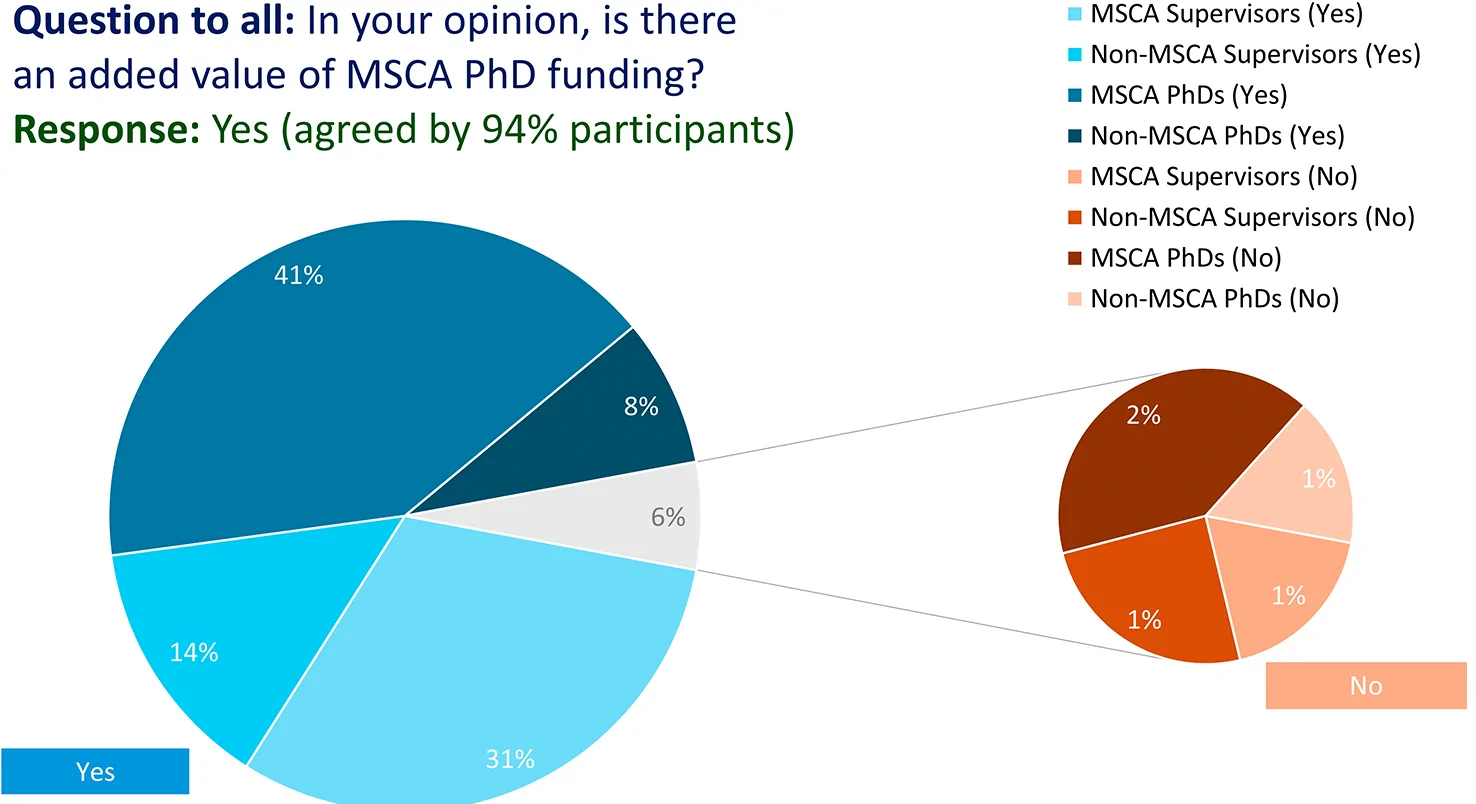
Is there an added value of MSCA PhD funding?.

Some of the most frequent terms used by the participants to elaborate on the added values of the ITN and DN include network, networking, internationality, interdisciplinarity, intersectorality, collaboration, funding, experience, salary, mobility, skills, industry, prestige, career etc. ( Figure 21).

**Figure 21.**
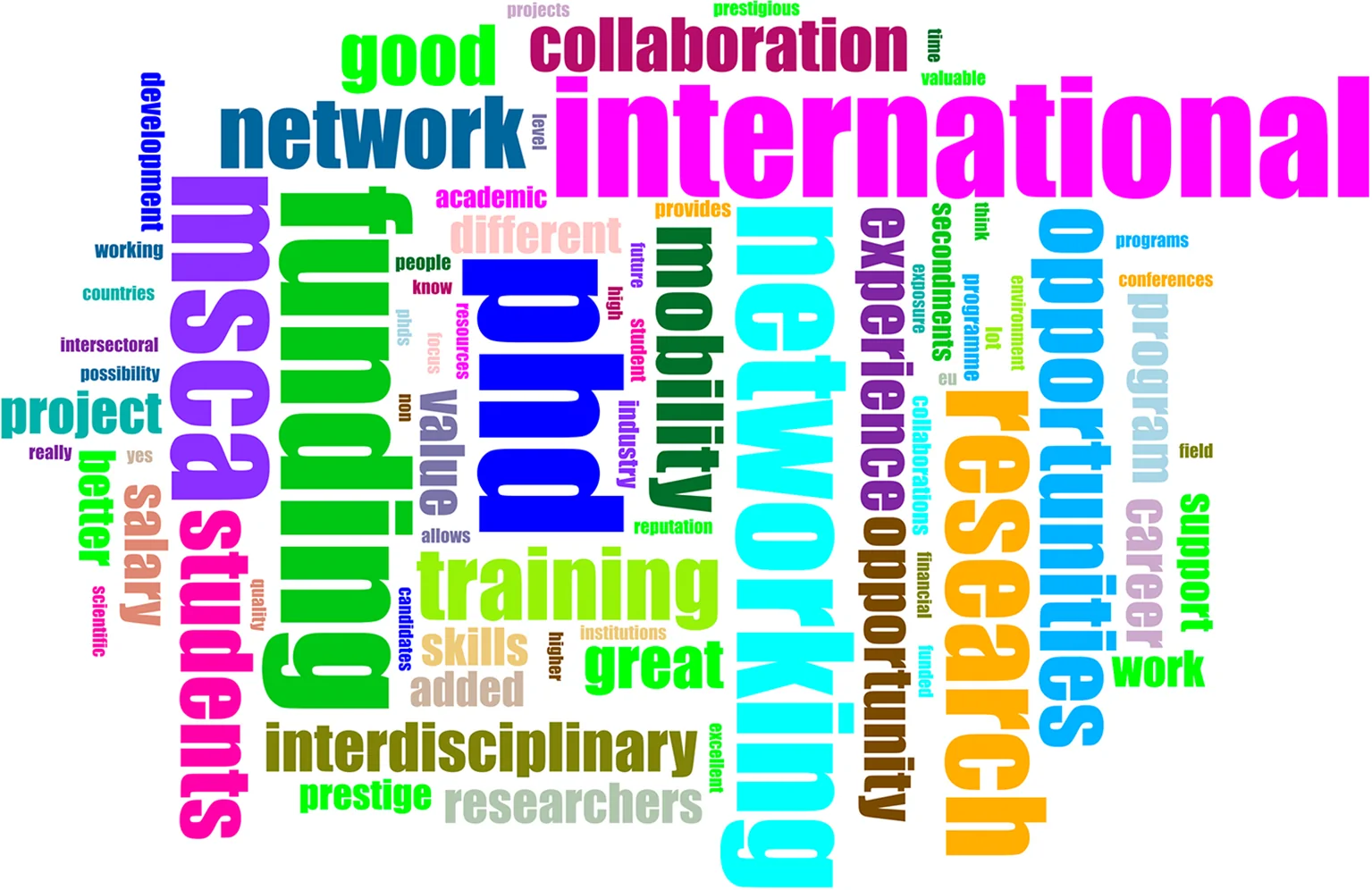
Added Values of MSCA ITN/DN, most frequently appearing words.

The findings of the survey as well as the interviews underscore that the added values of the ITN and DN lie in the effective execution of its networking frameworks, mobility across sectors, countries and disciplines, and improved working conditions which, as a large majority of the participants have indicated, enhance research, training, career development and employability. This is further confirmed by one of the MSCA supervisors in the interview who notes:

“You see, every euro that is invested makes an early-stage researcher better. And at the end of their three-year journey, you see just how much they have grown and how much their life has changed and how much their research has improved because of the Marie [Sklodowska] Curie network.” – MSCA Supervisor (Interview).

68% of the MSCA supervisors (20% more than non-MSCA) participating in the survey agreed that there are major differences between the MSCA and non-MSCA PhD programmes ( Figure 22) which are listed below.

**Figure 22.**
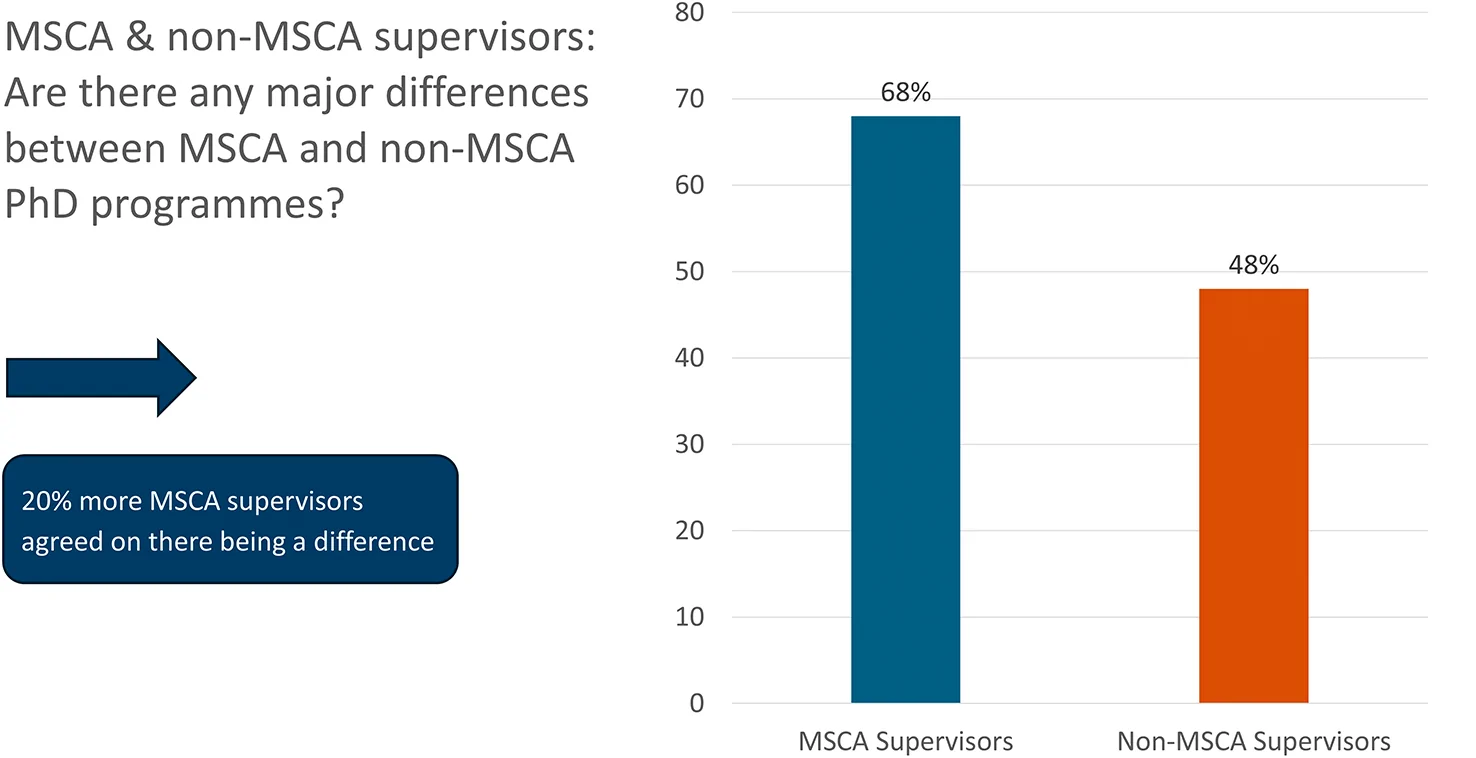
Yes, there are major differences between MSCA and Non-MSCA PhDs.

Some of the main strengths of the MSCA ITN and DN (compared to non-MSCA PhDs), as agreed by the MSCA and non-MSCA supervisors, include networking opportunities, triple “i” mobility, secondments, interdisciplinary and intersectoral supervision, career development, transferable skills training and training quality ( Figure 23).

**Figure 23.**
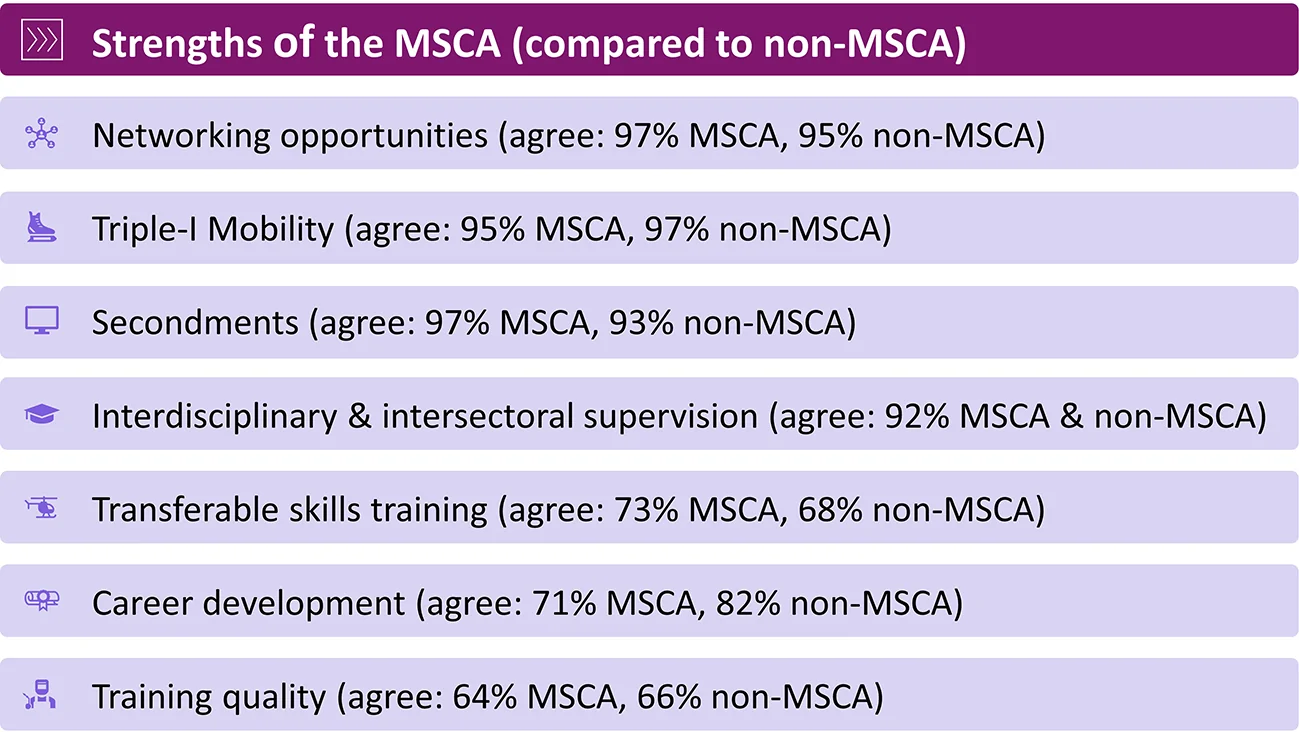
Supervisors’ opinion on the strengths of the MSCA compared to non-MSCA PhD programmes.

The results of the study also highlight that one of the added values of the MSCA ITN/DN programmes is rooted in their focus on mobility and networking. The MSCA ITN/DNs not only strengthen individual researchers but also fosters a robust and interconnected community of scholars across Europe and beyond. While mobility, networking, and collaboration are recognised components of most doctoral training frameworks, the MSCA ITN and DNs distinguish themselves especially by operationalising these elements effectively through its mandatory mobility, excellent training programme, structured network activities, and comprehensive support infrastructure. As one MSCA supervisor noted in the survey, “Working as a ‘class’ of PhD students on a common theme fosters great collaboration and peer learning opportunities”, an experience largely absent from many non-MSCA doctoral programmes.

The ITN and DN’s highly acclaimed provision of international mobility not only helps train MSCA fellows, but the networking opportunities and diverse cultural experiences that accompany it also provide the incentives required for Europe to retain as well as re-integrate talents within Europe. ^11, 14, 34^^,^ ^xiii^ Although several national initiatives may have a similar structure as MSCA demonstrating their structuring effect, they are widely diverse as they often operate with a rather limited scope with funding and/or training guidelines tied to the respective home countries. ^42^ The MSCA programme bridges this gap by promoting cross-border collaborations and fostering a harmonised and globally impactful research community. Furthermore, the international dimension of the MSCA ITN and DN not only promotes geographical mobility of the fellows but also increases higher intersectoral cross-fertilisation of knowledge as well as interdisciplinary collaborations.

### 4.8 Further Insights from the Survey

“All the Marie Curie programmes are generally seen as highly excellent, as very prestigious. So already from that perspective, being able to get such a grant, be it a post-doctoral fellowship, be it a doctoral network, this is always seen as something quite worthwhile and valuable. […] Additionally, the doctoral networks are designed in a way that includes many extra features and benefits. These go beyond what a regular doctoral candidate would typically have access to, making them even more beneficial.” – Stakeholder of doctoral research and training in Europe (Interview).

The role played by the MSCA ITN and DNs in supporting early-stage scientists coming from all over the world contributes to the EU’s goal to become the top global attraction for research and innovation as part of the Choose Europe for Science initiative. ^43^

The MSCA ITN and DNs have played an indispensable role in supporting research activities in Europe and beyond, which is demonstrated by the fact that 83% of the MSCA supervisors informed in the survey that they would not have been able to carry out their research project without the MSCA funding ( Figure 24).

**Figure 24.**
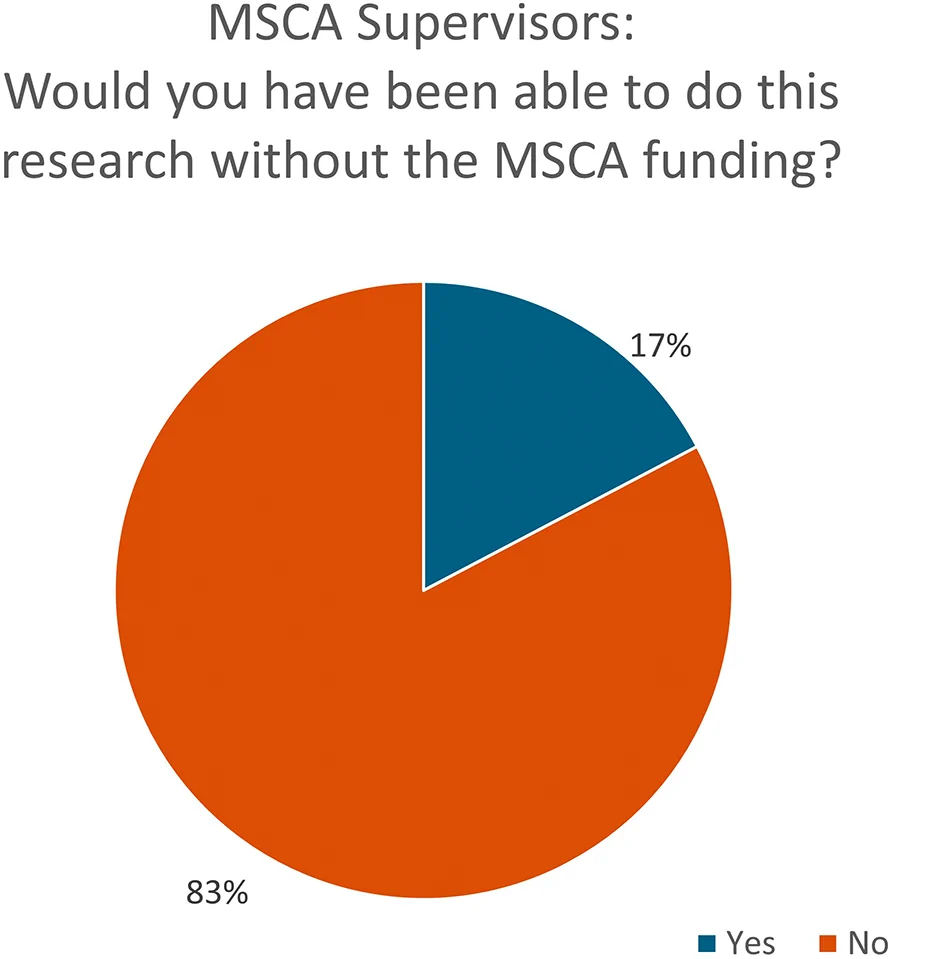
Would you have been able to do this research without the MSCA funding?.

The prestige and attractiveness of the MSCA ITN and DN funding is further resounded by non-MSCA supervisors, 79% of whom informed having applied or considered applying for MSCA ITN/DN funding. Although the global recognition and prestige of the MSCA as a whole is indisputable, ^11, 19, 28^ when it comes to the ITN and DN, the results of the survey show that it could still benefit from a boost in visibility.

A majority of the non-MSCA supervisors who have never considered applying for MSCA ITN or DN have listed the main reasons as lack of awareness about the existence of the programme, while not a few were dissuaded by other factors including complex programme requirements, complex application process, and low success rates ( Figure 25).

**Figure 25.**
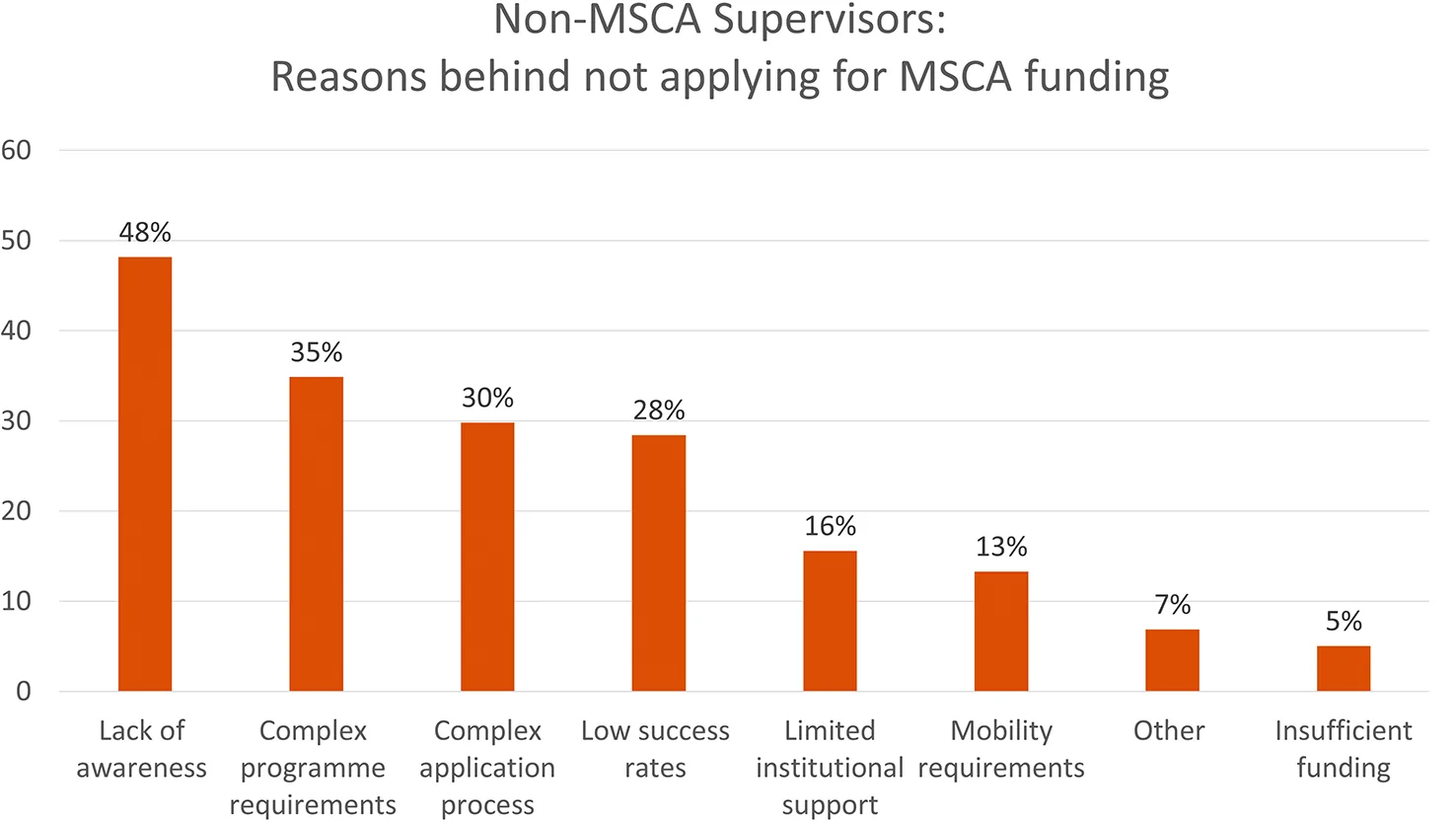
Reasons behind not applying for MSCA funding.

62% of the non-MSCA PhDs also indicated that they were unaware of the existence of the MSCA ITN (concluded in 2020) and DN programmes ( Figure 26).

**Figure 26.**
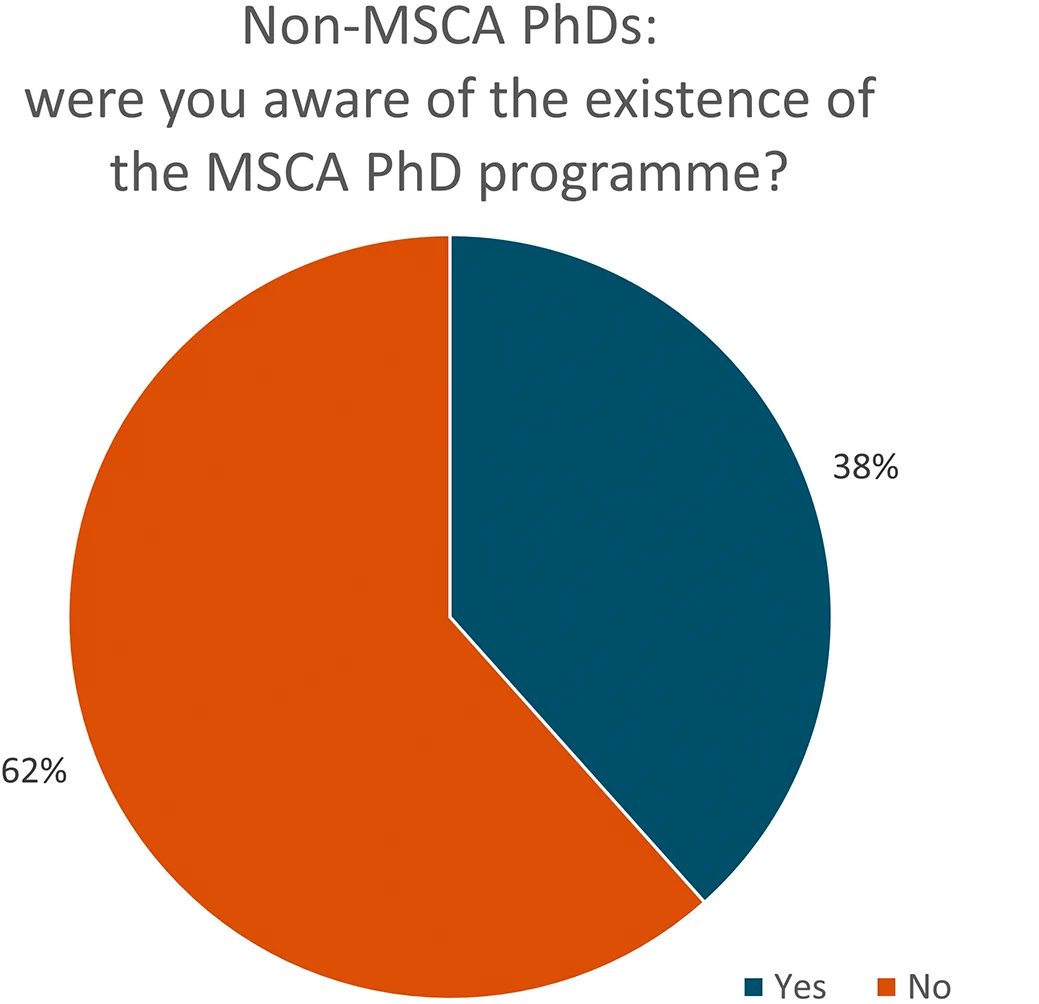
Lack of awareness about the MSCA ITN/DN.

A closer look into the geographic data on awareness about the ITN and DN indicates that over half of the non-MSCA participants across Europe are not well aware about the programme (see “adjusted percentage” in Table 5). ^xiv^ The non-MSCA participants who are less aware about the DN are mostly based in Denmark, France, Germany, the Netherlands, Poland, Spain, the UK etc. ( Table 5). For some countries, however, no generalisable comments could be made due to low sample size. These countries include Austria, Bulgaria, Croatia, Cyprus, Hungary, Lithuania, Luxembourg, Malta, Romania, and Slovakia ( Table 5).

**Table 5.** Non-MSCA participants lacking awareness about the MSCA ITN/DN (by country).

Percentage of non-MSCA participants (supervisors and fellows) who lack awareness about the MSCA ITN/DN by country			
Country	Raw percentage ^*^	Adjusted percentage ^**^	Number of non-MSCA respondents
Austria	76.5%	68.3%	17 ^***^
France	66.7%	65.0%	66
Iceland	100.0%	64.8%	2 ^***^
Netherlands	67.9%	64.4%	28
Czechia	69.2%	63.2%	13 ^***^
United Kingdom	64.3%	62.5%	42
Hungary	100.0%	61.6%	1 ^***^
Luxembourg	100.0%	61.6%	1 ^***^
Croatia	66.7%	60.3%	6 ^***^
Finland	61.5%	59.4%	13 ^***^
Poland	60.0%	59.0%	20
Spain	58.9%	58.8%	112
Germany	59.0%	58.7%	61
Denmark	59.3%	58.7%	27
Slovakia	50.0%	57.0%	2 ^***^
Lithuania	33.3%	55.5%	3 ^***^
Malta	33.3%	55.5%	3 ^***^
Italy	54.9%	55.3%	113
Ireland	50.0%	55.2%	10 ^***^
Norway	50.0%	55.2%	10 ^***^
Portugal	52.4%	54.9%	21
Romania	40.0%	54.8%	5 ^***^
Switzerland	52.0%	54.5%	25
Bulgaria	20.0%	54.3%	5 ^***^
Cyprus	44.4%	53.9%	9 ^***^
Greece	36.4%	51.6%	11 ^***^
Belgium	44.0%	50.5%	25
Sweden	35.7%	50.4%	14 ^***^

^*^ Global average of the sample = 57.7%.

^**^ K = 10 (Prior strength used for Bayesian smoothing. This value was chosen to stabilise estimates for low-sample groups while preserving observed variation in larger samples.)

^***^ Sample size not reliable.

The lack of awareness amongst non-MSCA participants emphasises on the need for promoting higher visibility of the DN. The same trend was highlighted in the interviews as well. For instance, as one of the current MSCA postdoctoral fellows whose PhD was not funded by the MSCA notes:

“When I was a master’s student and I was applying for the PhD programmes, I didn’t know about many grants that I now know, like those offered by the Horizon programme and the European Commission – and neither did my PI. Back then, I had very little knowledge about all the European grants available”. – MSCA PhD Fellow (Interview).

Other challenges noted by the participants include communication issues, visa and residence permit issues, as well as paperwork and reporting.

Although administrative issues are highlighted by all participants, a vast majority of the respondents received administrative support from their institutions at least to some extent, if not to a large or very large extent ( Figure 27).

**Figure 27.**
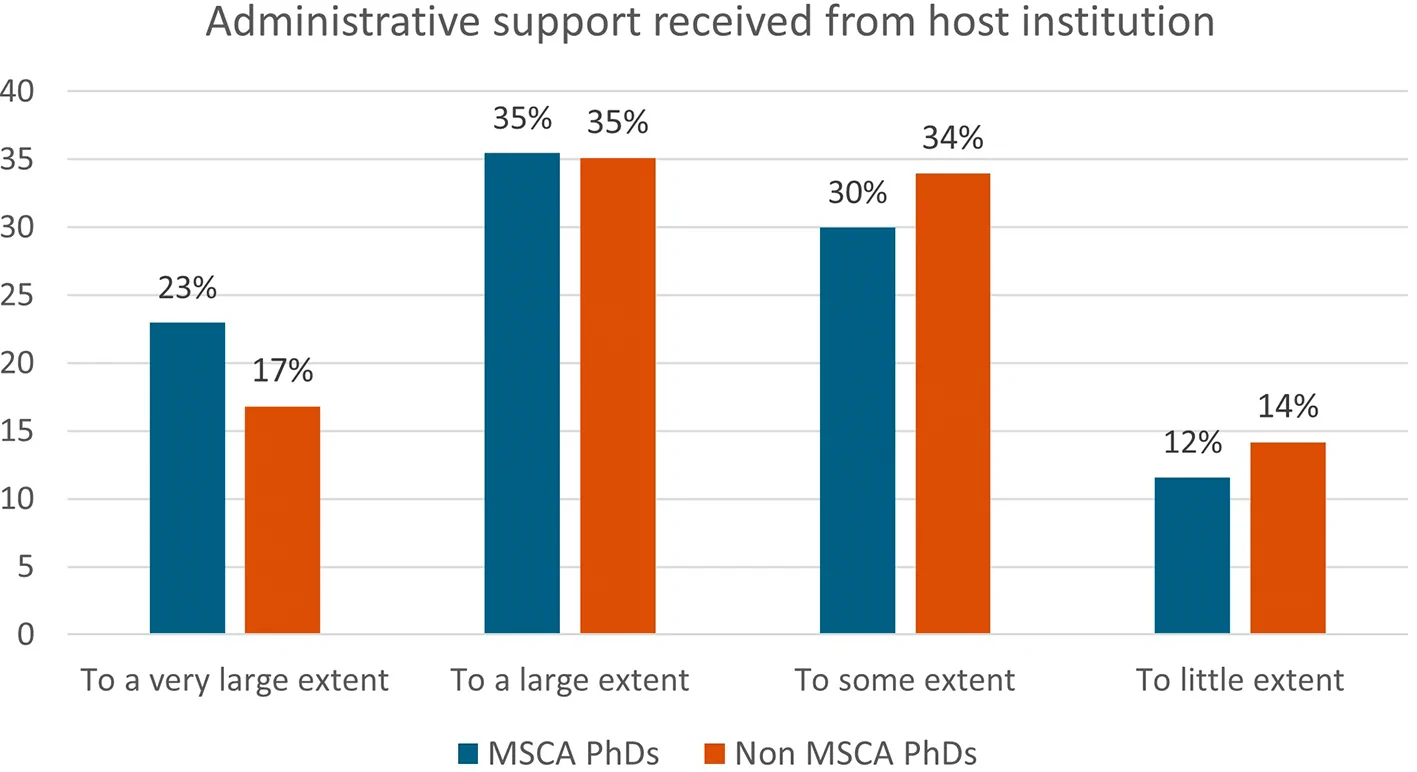
Administrative support received by PhD fellows from their institutions.

## 5. Discussion of the Main Impacts, Added Values and Pending Challenges

“The added value of MSCA PhD funding lies in its emphasis on mobility, interdisciplinary collaboration, generous financial support, and researcher development. It provides an unparalleled platform for PhD candidates to grow not just as researchers but as future leaders in both academia and industry.” – MSCA PhD Fellow (Survey).

The present study has strived to quantify the comparisons between MSCA and non-MSCA PhD programmes. To our knowledge, this represents the most robust and large-scale study conducted to date on the added value of MSCA ITN and DN projects. Despite the staggering number of participants in the survey, there are limitations due to the large variety of different doctoral programmes, the potential affinity of non-MSCA responders to MSCA participants and the influence of structuring effects that the MSCA have had over three decades on nationally funded doctoral programmes. Nevertheless, as the findings summarised above demonstrate, despite the structuring effects of the MSCA on national PhD programmes, the MSCA doctoral networks continue to excel through their offerings, impacts and added values compared to non-MSCA. The findings of this rigorous study can be used to further improve MSCA and infuse best practices to non-MSCA programmes. The study has examined the impacts and added values of the MSCA ITN/DN compared to non-MSCA across overarching areas including mobility, training, supervision, career development, and working conditions, as well as their broader structural effects on doctoral education in Europe and beyond. The key findings on the added values of the MSCA Doctorates, as well as the pending challenges, are summarised and discussed below.

### 5.1 Higher Triple “I” Mobility: International, Intersectoral, Interdisciplinary

MSCA ITN/DN participation is associated with markedly higher levels of international, intersectoral, and interdisciplinary mobility, with more researchers and supervisors gaining cross-border and cross-sector experience than non-MSCA counterparts. Fellows also report stronger positive impacts, including substantially greater industry exposure and improved outcomes in areas such as IPR strategy, sustainability, innovation, market insight, and collaborative activity.

The study reveals in particular that intersectoral mobility constitutes the most transformative dimension of the Triple “i” framework, generating substantially higher employment outcomes in non-academic sectors, with MSCA graduates securing such positions at three times the rate of non-MSCA counterparts. This finding carries significant implications for European research and innovation policy, as it demonstrates MSCA’s effectiveness in addressing a critical challenge within the European Research Area: the preparation of doctoral researchers for diverse career trajectories beyond academia. The 59% higher intersectoral mobility participation rate, combined with demonstrable career impacts, suggests that mandatory intersectoral elements and structured secondments create pathways that fundamentally reshape researchers’ professional identities and competencies. This transformation occurs not merely through exposure but through meaningful engagement with diverse fields, work practices, and innovation ecosystems. The finding that even non-Industrial Doctorate MSCA fellows achieve 36% higher intersectoral experience indicates that programme design, rather than mandate alone, successfully cultivates intersectoral engagement. From a policy perspective, this evidence supports scaling intersectoral mobility mechanisms across European doctoral education. This would require a more effective implementation, adequate support structures, reduced administrative barriers, and sufficient resources.

Skills development is also consistently superior, with enhanced social, technical, intercultural, and collaborative competencies and higher satisfaction with training across key areas. Additionally, both MSCA ITN/DN supervisors and researchers face fewer implementation barriers, including reduced challenges related to resources, adaptation, academic integration, communication, and navigating industry- and IPR-related processes.

### 5.2 Enhanced Career Outcomes and Employability

MSCA ITNs and DNs are associated with superior early-career outcomes, with 12% more fellows securing employment within one year of graduation, a threefold increase in placements outside academia, and 8% more venture beyond their core training fields. These advantages are reinforced by strong career development mechanisms embedded throughout MSCA training, including the substantial benefits of intersectoral mobility for career progression, expanded and durable professional networks, and structured, integrated career development support that collectively enhance long-term employability and career diversification, which is one of the crucial policy objectives of MSCA DN. This is further reinforced by the MSCA ITN/DN requirement to recruit Doctoral Candidates under an employment contract or other (equivalent) direct contract which enhances the researcher’s job security and stability, while full social security and in particular pension rights help improve the overall quality of life of researchers, allowing them to concentrate on their career development and research activities. Employers in both academia and industry are likely to view candidates with employment-based research experience as more skilled and ready for the workforce, given their familiarity with professional work environments and expectations. The same advantages stem from the requirement for full-time employment, as well as the almost mandatory exposure to the non-academic sector for all Doctoral Networks. Treating Doctoral Candidates as full-time employees demonstrates that the MSCA model has meaningfully and structurally shaped the professional framework of doctoral education in Europe and beyond.

### 5.3 Superior Training Quality and Supervision

MSCA DN programmes (including ITN) demonstrate strengthened supervisory and training quality, with 20% more fellows benefiting from supervisory teams, 15% more working under formalised supervision agreements, and overall supervision satisfaction rising since 2017. Training excellence is consistently highlighted, particularly in networking, transferable skills, and holistic researcher development. Key strengths include extensive networking opportunities, robust triple “i” mobility, high-quality secondments, interdisciplinary and intersectoral supervision, comprehensive career development support, and strong provision of transferable skills training. With the introduction of the MSCA Supervision Guidelines ^39^ in 2021, and their subsequent update in 2025, one could reasonably expect increased adherence among participating researchers and institutions to best practices and effective supervision frameworks. The introduction of the concept of a *supervision ecosystem*, which explicitly incorporates the institutional level, is likely to generate further structuring effects in this critical area of doctoral education.

### 5.4 Improved Working Conditions and Resources

MSCA ITN/DN actions foster strong working environments and project conditions. A significantly larger proportion of MSCA fellows (57% more) benefit from formal employment contracts, alongside improved social protection, with 14% more receiving social security coverage and 30% more expressing high satisfaction with their salary. Supervisors likewise highlight the programme’s strong financial support, with 39% more viewing project budgets as sufficient and 83% affirming that MSCA funding enabled research that would otherwise not have been possible. Together, these advantages underscore MSCA’s clear alignment with the European Charter for Researchers and its commitment to providing fair, secure, and highly supportive conditions for researchers and their teams.

However, the finding that 83% of supervisors could not have conducted their research without MSCA funding, alongside 39% higher budget sufficiency ratings, reveals a critical gap in alternative funding landscapes that MSCA addresses. The three-year funding duration concern, raised by MSCA fellows, Supervisors, and PIs, warrants examination within the context of optimising programme design to balance efficiency with adequate completion timeframes.

### 5.5 MSCA Structuring and Innovative Effects

The convergent recognition of MSCA’s added value by 94% of all respondents, including non-MSCA participants, provides compelling evidence of the programme’s normative influence extending beyond direct beneficiaries. This broad consensus, combined with quantifiable improvements in employment contracts (57% higher), social security coverage (14% higher), and salary adequacy (30% higher), demonstrates that MSCA functions not merely as a funding mechanism but as a catalyst for systemic transformation in doctoral education practices. However, the programme’s structuring effect operates through demonstration and aspiration rather than prescription: by establishing high standards for supervision quality, working conditions, and triple “i” mobility, MSCA creates benchmarks that influence institutional practices and national policies. This structuring effect aligns with Horizon Europe’s objectives of strengthening the European Research Area through harmonised excellence standards.

In parallel, the evaluation of research quality and impact within the MSCA framework has undergone a significant transformation in recent years. The European Commission has been a pioneer in integrating the principles of the Coalition for Advancing Research Assessment (CoARA), moving away from narrow reliance on traditional publication metrics toward a more comprehensive evaluation framework that encompasses diverse research outputs including datasets, software, public engagement activities, and policy contributions. ^7, 44, 45^ This transformation aligns with broader international movements, including the San Francisco Declaration on Research Assessment (DORA) and the Leiden Manifesto, which advocate for quality and openness over quantitative metrics alone. The MSCA programme increasingly recognises and values collaborative and interdisciplinary work, emphasising not only the societal and economic impact of research but also researchers’ skills, experiences, and potential for leadership and innovation. By facilitating international mobility and promoting the inclusion of underrepresented groups, the MSCA foster a diverse and globally connected research community in keeping with CoARA’s objectives. This also contributes to an ecosystem conducive to both tangible and non-tangible innovation within a more open, inclusive and innovative European research landscape, thus reinforcing the programme’s dual structuring and innovative effects.

## 6. Recommendations

Overall, the survey results indicate a high level of satisfaction among respondents. While these findings are encouraging and have clearly highlighted the added value of the MSCA ITN and DN programmes, several constructive suggestions emerged from the responses, providing an opportunity to build on existing strengths and identify specific areas where targeted improvements could further enhance outcomes. The positive survey outcomes confirm that current approaches are effective. To sustain this success and address minor gaps identified by respondents, a set of practical recommendations has been outlined below for the design and implementation of the programme under the new framework programme of the European Commission (FP10) which could further enhance the attractiveness and reach of MSCA Doctoral Networks, while reinforcing their contribution to excellence and innovation in doctoral training across Europe.

a. **Increase visibility and attractiveness**:
While the MSCA Doctoral Networks programme overall enjoys strong international recognition and credibility as suggested in the literature review, survey evidence suggests that the programme would benefit from further targeted communication efforts. The majority of non-MSCA supervisors who had not previously considered applying to MSCA ITNs or DNs cited limited awareness of the programme as the main barrier. In addition, 62% of non-MSCA PhD respondents indicated that they were not aware of the existence of the MSCA ITN/DN programme. This lack of awareness represents a critical challenge for maximising programme impact and ensuring equitable access to high-quality training opportunities across the European Research Area. This finding indicates that MSCA’s demonstrated added value, and transformative potential may not be reaching significant proportions of the target beneficiary community, potentially limiting programme uptake and geographic, disciplinary, and institutional diversity. The evidence suggests that enhanced strategic communication is required at the European level, including: strengthening dissemination channels through European research networks and professional associations, developing targeted outreach to underrepresented disciplines and institutions, creating accessible information resources that clearly articulate programme benefits, and mobilising the 94% of stakeholders who recognise MSCA’s value to share their experiences within their professional communities. The Commission can leverage existing frameworks such as National Contact Point networks and Marie Skłodowska-Curie Alumni Association channels to enhance visibility systematically. The celebration of the 30th anniversary of the MSCA programme could be an opportunity to boost the awareness and visibility of the programme which would support the wider uptake by potential applicants and host institutions, thereby reinforcing the programme’s visibility, reputation, and long-term impact.
b. **Increase duration of funding:**
While MSCA DNs offer three years fellowship as well as beneficial employment conditions, the survey findings indicate that this duration does not fully align with the structural requirements of many host institutions, where a four-year timeframe is often necessary to complete a PhD and represents the standard duration in several universities. In this context, 78% of MSCA fellows (28% more than non-MSCA respondents) reported that the three-year funding period poses challenges for timely PhD completion. This misalignment frequently leads MSCA ITN/DN fellows to seek alternative funding sources during the final phase of their doctorate.
In light of these findings, extending the individual fellowship duration from three to four years, alongside a corresponding extension of the overall project duration from four to five years, could enhance research continuity and improve the doctoral experience. Such an adjustment would reduce the need to secure additional funding in the final stages of the PhD, alleviate publication-related time pressures, and better align MSCA Doctoral Networks with prevailing institutional completion timelines.
c. **Increase amount of funding:**
Although the majority of MSCA supervisors and doctoral fellows reported that the MSCA salary is sufficient to cover living costs, a non-negligible share of respondents (19% of MSCA PhD fellows and 8% of MSCA supervisors) indicated that the salary is sufficient only to a limited extent. These findings suggest the need for further assessment.
Enhancing the attractiveness of MSCA Doctoral Networks, particularly for EU researchers and applicants from underrepresented countries, may require an adjustment of the living allowance. Such an adjustment could help address the comparatively lower participation of EU citizens (Source: CORDA), for whom MSCA remuneration is increasingly less competitive relative to national PhD salary levels. It would also alleviate pressures at institutional level in higher-income countries, where host organisations are often required to provide top-up funding through in-kind contributions.
A dedicated study is currently being launched to assess the competitiveness of MSCA DN salary levels and is expected to inform the appropriate level of funding required to maintain the attractiveness of the scheme.
In addition, in order to maintain, on average, a comparable number of funded researchers and to improve the programme’s overall success rate (which currently stands at approximately 10–12%), an increase in the total budget allocated to the programme should be considered.
d. **Strengthen Alignment between MSCA Doctoral Networks and National Doctoral frameworks to harmonise doctoral programmes in the European Education Area (EEA) and European Research Area (ERA):**
In terms of operational challenges, the survey highlighted administrative burdens and intercultural adaptation. Addressing these challenges would benefit from closer integration with national doctoral education frameworks, encompassing aspects such as admissions, supervision standards, and degree requirements. Harmonised procedures that ensure mutual recognition of national quality standards in MSCA evaluation and reporting, and vice versa, in the European Education Area (EEA) and the European Research Area (ERA) could create a mutually reinforcing structuring effect.
Ensuring that the design, standards, and practices of MSCA Doctoral Networks are closely aligned with national doctoral education systems would facilitate compatibility, interoperability, and the exchange of best practices across institutions and countries. This alignment will be greatly helped by the currently pursued efforts towards a European PhD label that meets common European requirements. As a gradual step towards a Joint European Degree, the European Degree Label is a new European initiative aimed at enhancing the recognition and quality of joint degree programs across Europe, while promoting student/researcher mobility and institutional cooperation.
e. **Administrative simplification:**
The survey findings and frequently reported factors demonstrate a perceived complexity of the application process and eligibility conditions, as well as complex programme requirements. Streamlining the application, evaluation and monitoring processes with a clear focus on excellence would facilitate the application process and result in efficiency gains for the applicants, while maintaining the objectives of the programme to raise the attractiveness and excellence of doctoral training in Europe. With regard to programme requirements and implementation, simpler and more flexible rules could help achieve the right combination to equip researchers with the right skills by allowing institutions and researchers to define themselves, based on the projects’ and researchers’ need, a more refined plan which would lead to improved employability and career prospects within and outside academia. Simplifying bureaucratic procedures, reducing paperwork and reporting requirements for the fellows and supervisors could enhance the supervision experience and better support research and training with minimal disruptions.

## 7. Conclusion

The findings of the study evidently demonstrate that the MSCA effectively advance the strategic objectives of the Horizon 2020 and Horizon Europe framework programmes by fostering research excellence, promoting researcher mobility and career development, strengthening international and intersectoral collaboration, and building a more integrated and competitive European Research Area.

These characteristics collectively position MSCA ITN and DN as exemplary research and training programmes fostering excellence and innovation in doctoral training at the global level, while the identified challenges, particularly regarding programme visibility, funding duration and project complexity, provide clear directions for continued programme evolution and enhancement.

Targeted simplification efforts could therefore help mitigate some of the identified challenges. Future studies could be conducted on a regular basis to monitor developments over time and could benefit from using automated tools to track the researchers’ career development. Finally, reaching consensus on indicators of doctoral programme quality would support a more systematic comparison of different doctoral models and facilitate the exchange of best practices.

## Ethics and Consent

Due to our affiliation with the European Commission, we do not have a regular institutional review board (IRB) or ethics committee like academic institutions. Instead, we adhere to the European Commission’s standards for research ethics, which prioritise transparency, integrity, and the protection of personal data as outlined by the General Data Protection Regulation (GDPR). ^9^ Our research protocols reflect these values, ensuring participant confidentiality and informed consent through rigorous application of these principles. Participant wellbeing and standard research ethics of studies involving human participants have been paramount throughout the study. Ethical considerations have been integrated into the research design to ensure compliance with general ethical norms of research involving human participants. We have anonymised all interview responses and survey data, thus protecting participants’ identity and wellbeing. All participants were provided with a Data Protection Notice ^10^ summarising the purposes of the study, and their right to withdraw at any point of time without any justification. Additionally, written consent was obtained from the interview participants through a signed consent form to anonymise and process the data obtained for the purpose of the study. All data obtained have been processed ethically following GDPR guidelines and have not been used for any other purpose than the current study and the dissemination of its results.

## Declaration of Generative AI and AI-assisted Technologies in the Writing Process

During the preparation of this work the authors used GPT@EC (formerly GPT@JRC), European Commission’s internal AI tool to enhance the language and readability. After using the tool, the authors reviewed and edited the content and take full responsibility for the content of the published article.

## Data Availability

The study draws from, and analyses data derived by means of literature review, interviews and survey. The sources consulted during literature review are provided in the reference section below. However, the interview transcripts and survey responses generated and analysed during the current study cannot be sufficiently de-identified and therefore cannot be made publicly available, due to ethical consideration. The interview transcripts analysed in the article are text-based transcriptions of video recordings, and the survey responses are in excel table format including closed and open text responses. Due to the large nature of the dataset including open responses, it is not possible to sufficiently deidentify personal information from all aspects of the data. Furthermore, the GDPR-compliant ^9^ Data Protection Notice ^10^ communicated to the participants does not explicitly allow the usage of the data beyond the purpose of the current study and its dissemination, and therefore, cannot be disclosed. Wherever necessary, these datasets have been complemented by data from the European Commission’s internal database, CORDA which cannot be made publicly available for the same reasons listed above. The said data could potentially be consulted and conditionally made available upon reasonable request. For this, please contact the corresponding authors at REA-HORIZON-MSCA-DN@ec.europa.eu. The questions guiding the interviews and the survey questionnaires have been made available on *Zenodo* under Creative Commons Attribution 4.0 International license (CC-BY 4.0) facilitating the replicability of the study. Links to all extended data are provided below. Bitsakis et al. Interview Questions: Study on the Added Value of MSCA Doctorates. *Zenodo* https://doi.org/10.5281/zenodo.18784047. ^46^ Bitsakis et al. Survey Questionnaire for PhDs: Study on the Added Value of MSCA Doctorates. *Zenodo* https://doi.org/10.5281/zenodo.18787279. ^47^ Bitsakis et al. Survey Questionnaire for PIs and Supervisors: Study on the Added Value of MSCA Doctorates. *Zenodo* https://doi.org/10.5281/zenodo.18787420. ^48^ Microsoft Office Excel was predominantly used to analyse the data. The web-based application Voyant Tools was used for generating a word cloud ( Figure 21) referred to in the article. Voyant Tools can be used for similar purpose by visiting this link: https://voyant-tools.org/. ^49^

## Footnotes

^i^
Established as Marie Curie Actions in 1996, the programme was rebranded as Marie Skłodowska-Curie Actions in 2014.

^ii^
The focus of this study is on Innovative Training Networks (ITN) under Horizon 2020 (2014–2020), and Doctoral Networks (DN) under Horizon Europe (2021–27).

^iii^
Interdisciplinarity refers to the integration of information, data, techniques, tools, perspectives, concepts or theories from two or more scientific disciplines. The term discipline refers to the first level of MSCA keywords, https://rea.ec.europa.eu/system/files/2021-10/MSCA%20Keywords.pdf.

^iv^
Intersectorality refers to the mobility between academic and non-academic sector. Non-academic sector includes any socio-economic actor (e.g., SMEs, industry, hospitals, museums, NGOs, and other societal actors) not included in the academic sector and fulfilling the requirements of the Horizon Europe Rules for Participation, https://eur-lex.europa.eu/EN/legal-content/summary/horizon-europe-the-framework-programme-for-research-and-innovation-laying-down-its-rules-for-participation-and-dissemination.html.

^v^
In the context of survey participants, the term “supervisor” has been used to refer to both Principal Investigators (PIs) and supervisors, for the sake of brevity.

^vi^
As of 2025, only DN2021 projects are now entering their second period whereas the rest of the DN projects are still in their initial phase where not many secondments take place.

^vii^
Special needs allowance was introduced in 2019.

^viii^
Long-term leave allowance was introduced in 2021.

^ix^
Intergenerational educational mobility is a key dimension of social mobility, referring to the extent to which educational attainment is transmitted between parents and their children. As Valentini explains, it reflects the relationship between parental education levels and the educational outcomes of the next generation, and serves as an indicator of equality of opportunity within a society. High levels of intergenerational educational mobility indicate that individuals are able to attain higher levels of education than their parents, while low mobility points to the persistence of educational advantage or disadvantage across generations.

^x^
The results shown in the table have been generated by calculating an adjusted percentage using the Bayes’ theorem, for which a k-value of 30 was added to all variables to adjust the raw percentages considering that for some countries the sample size was below 20, whereas many had close to 100 or significantly more participants. No generalisable comments can be made for the countries where the sample size was below 20. Furthermore, the data may not accurately represent countries where institutions top up the salaries of MSCA fellows because these salaries fall below the national rates (for example, Denmark, Finland etc.).

^xi^
It is noted that there were significantly fewer participants (n < 60) in the survey from MAT and ECO panels compared to others which had more than five times more participants. This is representative of the proportion of ITN/DN projects funded under those panels, receiving less applications.

^xii^

The European University Association (EUA) Doctoral Education Programme, the Bologna Process, Networks and Consortia, the Coimbra Group, MSCA Programmes, European Research Area (ERA) etc. are some of the notable examples of efforts at harmonising and standardising higher education.

^xiii^
According to the 2023 annexes of a study by the European Commission: DG RTD and others, 65% of third country nationals (IF, ITN and COFUND actions), who were hosted in EU Member States, were employed in a Member State two years after the end of their fellowship. Third country ITN fellows are more likely to stay in Europe than IF and COFUND fellows: for ITN, the share of third country nationals who stayed in Europe is 74%, while for IF and COFUND the shares are 55% and 44% respectively. As per the 2023 report by the European Commission: DG RTD and others, the MSCA not only contributed to retaining a substantial number of European researchers in Europe, but also helped attract both European and third-country researchers living abroad.

^xiv^
The results shown in the table have been generated by calculating an adjusted percentage using the Bayes’ theorem, for which a k-value of 10 was added to all variables to adjust the raw percentages considering that for some countries the sample size for this specific question was below 10.
