# Supplementary material for: Added value of MSCA doctorates: A mixed-methods study
Source: Open Res Eur. 2026 Sep 8;6:227. Originally published 2026 Jul 2. [Version 2] doi: 10.12688/openreseurope.23294.2 (PMC13650323; doi:10.12688/openreseurope.23294.2)
Supplement: Peer review history

# Reviewer report: Reviewer response for version 2

**DOI:** 10.21956/openreseurope.27006.r80663

**Published:** 2026-10-08

**Authors:** Thomas Wintgens

**Affiliations:** 1 RWTH Aachen University, Aachen, Germany

I approve the revision and thank for the detailed response.

If applicable, is the statistical analysis and its interpretation appropriate?

Partly

Is the study design appropriate and is the work technically sound?

Yes

Is the work clearly and accurately presented and does it engage with the current literature?

Yes

Are the conclusions drawn adequately supported by the results?

Yes

Are sufficient details of methods and analysis provided to allow replication by others?

Partly

Are all the source data and materials underlying the results available?

Yes

Reviewer Expertise:

NA

I confirm that I have read this submission and believe that I have an appropriate level of expertise to confirm that it is of an acceptable scientific standard.

# Reviewer report: Reviewer response for version 2

**DOI:** 10.21956/openreseurope.27006.r80662

**Published:** 2026-09-23

**Authors:** Inma Alvarez

**Affiliations:** 1 The Open University, Milton Keynes, UK

I am writing to let you know that I checked the revisions and I am satisfied on how they have been addressed.

If applicable, is the statistical analysis and its interpretation appropriate?

Yes

Is the study design appropriate and is the work technically sound?

Yes

Is the work clearly and accurately presented and does it engage with the current literature?

Partly

Are the conclusions drawn adequately supported by the results?

Yes

Are sufficient details of methods and analysis provided to allow replication by others?

Yes

Are all the source data and materials underlying the results available?

Yes

Reviewer Expertise:

NA

I confirm that I have read this submission and believe that I have an appropriate level of expertise to confirm that it is of an acceptable scientific standard.

# Reviewer report: Reviewer response for version 1

**DOI:** 10.21956/openreseurope.25201.r76939

**Published:** 2026-07-23

**Authors:** Thomas Wintgens

**Affiliations:** 1 RWTH Aachen University, Aachen, Germany

The presented study analyses comprehensively the competence and career development related impact of MSCA doctorates. It is very timely and relevant to analyse the impacts of such an important funding programme as MSCA doctoral networks. The study builds on different methods including a literature review, qualitative interviews and a survey among MSCA-participants and participants to other doctoral programmes (non-MSCA participants). The combination of the chosen methods is very appropriate for the scope of the study.

 The survey has achieved a very high participation which provides a comprehensive basis for the analysis. It is recommended to provide some more methodological details e.g. related to the selection of interview partners within the group of survey participants and the type of non-MSCA programmes covered in the survey. The claim that the sample size related to the non-MSCA participants in the survey seems reasonable but should be substantiated, e.g. by addressing key criteria for representativeness. The assessment criteria cover a set of important impact categories such as international and inter-sectorial mobility as well as career opportunities of the graduates and the funding conditions, relevant both to the trained researchers and the hosting institutions. However, it should be acknowledged that there are also other important criteria such as scientific output.

 The result section (4. Findings) provides a very comprehensive set of criteria being analyzed. Generally, the section could be more concise. It is recommended to shorten the section, possibly some figures could be put into an annex. While most results refer to the survey data, some excerpts from the interviews complement the analysis. A more systematic overview on the interview synthesis could be beneficial. Some more detailed comments:

- Figure 5: It means that a 100% employment rate is achieved in both groups? After which duration beyond the project?
- Table 1: Information is derived from survey responses? Which indicators have been used to assess economic and environmental impact as well as sustainability?
- Figures 11-13 could probably be placed in an annex. Just short text summary.
- Information contained in Figures 22, 24, 26 could be presented in a consolidated form
- Table 5 could be put in an annex.
- Section 5 should include a section on the Limitations of the study. Did the survey and interviews also include recommendations provided by the participants? Those could be summarized as well.

If applicable, is the statistical analysis and its interpretation appropriate?

Partly

Is the study design appropriate and is the work technically sound?

Yes

Is the work clearly and accurately presented and does it engage with the current literature?

Yes

Are the conclusions drawn adequately supported by the results?

Yes

Are sufficient details of methods and analysis provided to allow replication by others?

Partly

Are all the source data and materials underlying the results available?

Yes

Reviewer Expertise:

NA

I confirm that I have read this submission and believe that I have an appropriate level of expertise to confirm that it is of an acceptable scientific standard, however I have significant reservations, as outlined above.

# Response

**Published:** 2026-08-25

**Authors:** Malay BERA

**Responses to reviewer #2** **Authors' response:** We would like to thank the reviewer for the detailed comments, and note that we have incorporated their suggestions in the revised version. Our responses to the reviewer's comments are given below explaining the revisions.

**Reviewer #2: **The survey has achieved a very high participation which provides a comprehensive basis for the analysis. It is recommended to provide some more methodological details e.g. related to the selection of interview partners within the group of survey participants and the type of non-MSCA programmes covered in the survey. The claim that the sample size related to the non-MSCA participants in the survey seems reasonable but should be substantiated, e.g. by addressing key criteria for representativeness. The assessment criteria cover a set of important impact categories such as international and inter-sectorial mobility as well as career opportunities of the graduates and the funding conditions, relevant both to the trained researchers and the hosting institutions. However, it should be acknowledged that there are also other important criteria such as scientific output.  

**Authors' response:** It has been clarified under methodology that the interview participants were selected based on a pool of referrals from within REA and the initially identified candidates. The selection criteria aimed for balance of gender and experience across groups, and final participation depended on the availability of the candidates. Additional invitations were sent to compensate for non-response and refusals. Non-MSCA participation targeted individuals with experience in pursuing or supervising a PhD in Europe, but not connected to ITN and DN. Geographical representativeness could not be guaranteed for interviews due to the limited sample size, however, the wide geographical coverage of the survey compensates for this lack in overall findings presented. In line with the reviewer's suggestions, it has been acknowledged in the revised version that there are other criteria as well such as scientific output. However, they have not been taken into account in this study as the DN is still ongoing (and therefore, assessing that crriteria would yield incomplete results), whereas a study on the innovative dimension and output of the ITN is already available by Ioannis Bitsios and others published in 2023. [1]

**Reviewer #2: **The result section (4. Findings) provides a very comprehensive set of criteria being analyzed. Generally, the section could be more concise. It is recommended to shorten the section, possibly some figures could be put into an annex. While most results refer to the survey data, some excerpts from the interviews complement the analysis. A more systematic overview on the interview synthesis could be beneficial.  

**Authors' response:** Interviews have been incorporated within the main text addressing the suggestions from both reviewer #1 and #2.  

**Reviewer #2: **Figure 5: It means that a 100% employment rate is achieved in both groups? After which duration beyond the project?

**Authors' response:** No, 92% of MSCA and 80% non-MSCA are employed within a year of completion of PhD, more details are provided in Figure 16 and corresponding analysis. Clarification has been added in the revised version, along with cross-reference to Figure 16.

**Reviewer #2: **Table 1: Information is derived from survey responses? Which indicators have been used to assess economic and environmental impact as well as sustainability?

**Authors' response:** Yes, the information has been derived from survey responses, and the same has been indicated in the updated table. Both environmental impact and sustainability, in this context, are to be understood in terms of the MSCA Green Charter (See quote below, reference has been added in the revised version as well). The information presented reflect the participants’ opinion in response to the question: In which fields did you experience a positive impact stemming from your triple “i” mobility experience? The question was asked separately for all three aspects of triple “i” mobility and have been synthesised into one table to avoid repetition. Only the fields in which the proportion of MSCA respondents recognising an impact is at least 10 percentage points higher or lower than that of non-MSCA respondents are presented to assess the added value.  

***Quote from MSCA Green Charter on environmental impact and sustainability:***

*“The European Commission is committed to tackling climate, environmental and biodiversity challenges, in line with the EU Green Deal **[2]**, the United Nation’s 2030 Agenda **[3]** and the Sustainable Development Goals **[4]**. As an organisation, the European Commission is also determined to act as a front-runner in the transition towards a climate neutral society, by setting out an ambitious and realistic plan to achieve climate neutrality by 2030 **[5]**.*

*In light of the role of the MSCA programme supporting the future generations of researchers at the highest level, the MSCA Green Charter lays down a set of non-binding guiding principles that promotes the mainstreaming of environmental considerations in all aspects of research planning and implementation, throughout the life cycle of a project. The Charter seeks to help reduce environmental harm caused or induced by MSCA-funded projects, to raise awareness of environmental sustainability, and to serve as a catalyst in promoting sustainable practices in research.” **[6]***

**Reviewer #2: **Figures 11-13 could probably be placed in an annex. Just short text summary.

**Authors' response:** We appreciate the suggestion to move Figures 11–13 to an annex, and we considered this option. However, in the end, it was decided to retain the figures in the main body, as they provide useful visual detail and support the discussion of the findings directly. We consider that removing them from the main text would make the relevant findings less accessible to the reader, especially because then it will have to be added as extended data as it is not possible to host annexes in ORE.  

**Reviewer #2: **Information contained in Figures 22, 24, 26 could be presented in a consolidated form

**Authors' response:** We considered the suggestion to present the information from these figures in a consolidated form as well. However, the three figures illustrate distinct findings within the overall discussion, and Figures 24 and 26 are separated by Figure 25, which addresses a different topic. The current order and presentation follow the logic of the discussion and are important for maintaining the flow of the section. We have therefore retained the figures in their current form and location.  

**Reviewer #2: **Table 5 could be put in an annex.

**Authors' response:** We considered the suggestion to move Table 5 to an annex. However, we have retained it in the main body because the information presented in the table is directly relevant to the discussion and allows the reader to consult the underlying results alongside the text. We consider that keeping the table in the main body provides greater clarity and accessibility instead of linking them to extended data on another platform since it is not possible to host annexes in ORE.  

**Reviewer #2: **Section 5 should include a section on the Limitations of the study. Did the survey and interviews also include recommendations provided by the participants? Those could be summarized as well.

**Authors' response:** Incorporating reviewer #2's suggestion, limitations have been noted at the end of section 5.  As clarified within the revised version, the recommendations section has also been prepared on the basis of overarching recommendations from both the interviews and the survey participants. These recommendations from both components of the study largely complement each other and therefore, no separate section was prepared for the two to avoid repetitions. Specific nuances from the interviews have been incorporated in the study thematically and may be consulted in the revised version.  

**References**

 [1] Bitsios I, Martone F, Ricci R, et al.: The Innovative Dimension of the Research Training Programmes under H2020-MSCA-ITN1: A Methodological Approach to Track, Measure and Analyse Innovative Aspects and Provide Policy-Feedback Conclusions. *F1000Res*. 2023;12:1020. https://doi.org/10.12688/f1000research.138482.2.   [2] Communication from the Commission to the European Parliament, the European Council, the Council, the European Economic and Social Committee and the Committee of the Regions. *The European Green Deal *(COM(2019) 640 final): https://eur-lex.europa.eu/legal-content/EN/TXT/?uri=CELEX :52019DC0640.   [3] United Nations: *Transforming our world: The 2030 Agenda for Sustainable Development.* https://sdgs.un.org/2030agenda.   [4] United Nations: *The 17 goals.* https://sdgs.un.org/goals.   [5] European Commission: *Greening the European Commission.* https://commission.europa.eu/about/service-standards-and-principles/modernising-european-commission/greening-european-commission_en.   [6] European Commission: DG EAC: *Marie Skłodowska-Curie Actions Green Charter – Guidance material for researchers and research managers*. Publications Office of the European Union;2025. https://data.europa.eu/doi/10.2766/3901124.

# Reviewer report: Reviewer response for version 1

**DOI:** 10.21956/openreseurope.25201.r76933

**Published:** 2026-07-20

**Authors:** Inma Alvarez

**Affiliations:** 1 The Open University, Milton Keynes, UK

**General**

 The introduction of the paper offers useful, detailed background information about the nature of the Marie Skłodowska-Curie Actions and Networks. It highlights the innovative aspects of this doctoral programme which aims at establishing meaningful connections between academia and the real world. The study looks at the intersection of three key dimensions of career mobility for doctoral and postdoctoral researchers (international, intersectoral, and interdisciplinary).

 The research design presents a comparative study of both MSCA and non-MSCA which sheds light on the effectiveness of the added value of the programme. While data from each of those distinct groups was not similar in terms of numbers, the comparison is still made on the basis of large amounts of data. This means a likelihood of having reached saturation of information and making the data more representative than claimed. The authors are however careful and highlight this quantitative difference as a limitation.

 A couple of general comments. Firstly, there is considerable emphasis on the quantitative results, and the analysis could be strengthened with the nuances presented in the qualitative data. For instance, by critically commenting on the aspects that were perceived less favorable. Secondly, there is some repetition of the information which could be avoided. Mainly in the Methodology and in the Findings and Discussion.

**Methodology**

 Regarding the methodology, a few minor additions to the information would add to the rigor of the reporting. While there is some repetition with respect to the ethical considerations (e.g. wellbeing of participants, anonymization), some details of the process have not been noted. For instance, it will be useful to add an explicit mention about how and how much data was collected (there is no mention of the platform used, assuming all was done online, or the duration/extension of the interviews and survey.)

 The aims of the data collection as well as the methodological limitations are clearly stated. However, it would enhance the clarity of the methodology if participation in the study was shown on a table with both numbers and percentages and it included all data collected, including the interviews as well. At the moment, in Figure 2 only shows the percentages of survey participants and in a redundant manner, i.e. in two different formats (a circle and a table). Also, importantly, greater granularity regarding the personal characteristics (such as sex, age, disability) of the participants, particularly of doctoral and postdoctoral researchers would have provided insights on the extent of the equity of the programme.

 The sentence “The survey questionnaire was specifically designed to hear the voices of the participants” reads a bit odd since surely this was also the case with the interview questions. Perhaps this comment could be made regarding both research tools and just deleted.

 The study explains the main details of the ethical approach (anonymity, confidentiality, withdrawal, etc.). Regarding consent, it indicates that written consent was obtained from the interviewees, but it is unclear whether it was also sought for respondents to the survey. This should be explicitly stated. The authors may want to consider whether the ethical statements need to be repeated at the end of the paper. Perhaps a cross-reference between this section and the final statement could be added

**Literature review**

 The literature review acknowledges sources of evidence between 2011 and 2025 with no rationale for selecting this particular period of time. It would be good to know why this timeframe was decided given that the MSCA was launched in 2014 and its predecessor MCA started in 1996.

 There is ambiguity in the first paragraph of section 3.2: “Although historically, there has been a gap in supporting comprehensive skills development among **PhD holders** in…. high standards in **doctoral education**”. Is this referring to postdoctoral training or during doctoral studies or both?

 The Methodology claims that the literature review was based on “110 existing literature items”, but there is also a statement on Data Availability that reads, “The sources consulted during literature review are provided in the reference section below”; the list displays 49 references, how does this relate to the 110 papers? The actual text in the section of the literature review refers to mostly grey literature regarding the programme without acknowledging other empirical studies on MSCA or other kinds of doctoral and postdoctoral training. For instance: [Ref 1 & 2]

 It would be helpful if the reporting of the sources consulted is revised for consistency and coherence purposes.

 The literature review is well-structured into themes of interest regarding the programme. It indicates clearly issues and trends and explicitly positions the study within current knowledge.

**Findings**

 The findings section presents rich data in the text and in relevant Figures. It is unclear whether Figures are based on both the interview and the survey or just the survey. A clarification to this regard should be added, for instance, to the introduction of section 4 or reflected in the Figure captions.

 In relation to the above, some statements need precision. For example, the text reads, “As one MSCA supervisor eloquently stated, the intersectoral dimension (and triple “i” mobility as a whole) of MSCA ITN/DN programmes fosters the emergence of excellently trained young scientists, poised to bridge the gap between industry and academia with their diverse skill sets.” There are no quotation marks or indication whether the statement was provided in the interview or the survey.

 The findings offer critical insights with respect to the consistently enhanced mobility reported by those in the programme compared to their peers not participating in it. It would be useful to see more qualitative data in the form of direct or indirect quotes from the participants. This would help support some of the authors’ interpretations of the quantitative data. Also, the current selected quotes reflect positive comments; some of the issues flagged by the participants could be heard as well. One of the exceptions is the identification, analysis and commentary regarding funding awareness.

 The discussion around salary is appropriately contextualized with geographical locations as per Table 4 where the cost of living is used as a benchmark. Figure 12 displaying supervisors’ impressions on budgets per subject together with the information regarding the variations in the duration of doctoral programmes prompt useful funding recommendations.

**Discussion**

 This section mostly synthesizes the findings, more critical commentary on their significance against the background of previous knowledge would strengthen the argument.

**Recommendations**

 This section is very helpful in practical terms and is supported by the data. There is only a small issue with the initial paragraph which only refers to the survey. This gives the impression of the authors valuing more the quantitative data. Were the interviews insignificant for the recommendations?

If applicable, is the statistical analysis and its interpretation appropriate?

Yes

Is the study design appropriate and is the work technically sound?

Yes

Is the work clearly and accurately presented and does it engage with the current literature?

Partly

Are the conclusions drawn adequately supported by the results?

Yes

Are sufficient details of methods and analysis provided to allow replication by others?

Yes

Are all the source data and materials underlying the results available?

Yes

Reviewer Expertise:

Higher education; postgraduate studies; Equity, diversity and inclusion; Language education

I confirm that I have read this submission and believe that I have an appropriate level of expertise to confirm that it is of an acceptable scientific standard, however I have significant reservations, as outlined above.

## References

[1] : Innovative training networks: overview of the Marie Skłodowska-Curie PhD training model. *FEMS Microbiology Letters* .2018;365(19) : 10.1093/femsle/fny207 10.1093/femsle/fny207 PMC614091530239741

[2] : Dynamics of return mobility among experienced researchers. An analysis of determinants and impact of Marie Sklodowska-Curie actions. *Globalisation, Societies and Education* .2025; 10.1080/14767724.2024.24458581-15 10.1080/14767724.2024.2445858

# Response

**Published:** 2026-08-25

**Authors:** Malay BERA

**Responses to reviewer #1** **Authors' response:** We would like to thank the reviewer for the detailed comments, and note that we have incorporated their suggestions in the revised version. Our responses to the reviewer's comments are given below explaining how the comments have been incorporated into the revised version.

- *
**General**
*

**Reviewer #1:** A couple of general comments. Firstly, there is considerable emphasis on the quantitative results, and the analysis could be strengthened with the nuances presented in the qualitative data. For instance, by critically commenting on the aspects that were perceived less favorable. Secondly, there is some repetition of the information which could be avoided. Mainly in the Methodology and in the Findings and Discussion.

**Authors' response:** The analysis has been strengthened with nuances presented from the qualitative data. Interview quotes have been inserted on time constraints (4.4 social innovation & working environment), complex application process and programme requirement (4.8 further insights), salary levels (4.4 social innovation & working environment), mobility challenges and flexibility (4.1.3 challenges to triple "i" mobility), institutional challenges in maintaining international network (4.8 further insights), and institutional-level communication issues (4.8 further insights).  Repetition in methodology, findings and discussion have been removed as well.

- *
**Methodology**
*

**Reviewer #1:** Regarding the methodology, a few minor additions to the information would add to the rigor of the reporting. While there is some repetition with respect to the ethical considerations (e.g. wellbeing of participants, anonymization), some details of the process have not been noted. For instance, it will be useful to add an explicit mention about how and how much data was collected (there is no mention of the platform used, assuming all was done online, or the duration/extension of the interviews and survey.)

**Authors' response:** The methodology section has been revised to reflect that we did 34 interviews of approximately one hour long each. The interviews were done through Microsoft Teams, and the survey was launched on EU Survey which contained around 30 closed and open-ended questions (could be slightly more or less, depending on conditional questions), and took approximately 20 minutes to complete.

**Reviewer #1: **The aims of the data collection as well as the methodological limitations are clearly stated. However, it would enhance the clarity of the methodology if participation in the study was shown on a table with both numbers and percentages and it included all data collected, including the interviews as well.

**Authors' response:** A new table has been added in the methodology section reflecting the actual number of interview participants by category and gender (percentages have not been provided considering the relatively fewer number of interviews). Figure 2 has been revised as well, to reflect the actual number of survey participants along with percentages.

**Reviewer #1: **At the moment, Figure 2 only shows the percentages of survey participants and in a redundant manner, i.e. in two different formats (a circle and a table). Also, importantly, greater granularity regarding the personal characteristics (such as sex, age, disability) of the participants, particularly of doctoral and postdoctoral researchers would have provided insights on the extent of the equity of the programme.

**Authors' response:** Figure 2 has been updated to reflect the total number of survey participants, including the number of PhD graduates and ongoing fellows. Since the study was designed to focus on doctoral experiences of the participants, we cannot provide any details regarding postdoctoral researchers which was beyond the study’s scope. As for doctoral researchers, the personal characteristics data collected in the survey mainly included country, status of graduation, and employment. The sex of interview participants has been provided to ensure transparency, however, such data have not been collected from the survey participants due to the fact that although ITN calls have ended, DNs are still ongoing, and DN demographic data at this stage may not be comparable against ITN. Therefore, we have rather focused on experience level, i.e., PhD fellow and supervisor. However, the recommendation is well-noted, and a comment has been added under limitations in methodology and discussion.

**Reviewer #1: **The sentence “The survey questionnaire was specifically designed to hear the voices of the participants” reads a bit odd since surely this was also the case with the interview questions. Perhaps this comment could be made regarding both research tools and just deleted.

**Authors' response:** The paragraph containing this sentence has been deleted to remove redundancy.

**Reviewer #1: **The study explains the main details of the ethical approach (anonymity, confidentiality, withdrawal, etc.). Regarding consent, it indicates that written consent was obtained from the interviewees, but it is unclear whether it was also sought for respondents to the survey. This should be explicitly stated. The authors may want to consider whether the ethical statements need to be repeated at the end of the paper. Perhaps a cross-reference between this section and the final statement could be added

**Authors' response:** Although no written consent was taken from the survey respondents, a Data Protection Notice was shared with them in the email invitation as well as in the EU Survey form explaining GDPR compliance, voluntary participation, right to withdraw etc. All relevant details have been declared under Ethics and Consent section at the end of the article. The same has been tightened in the methodology section to remove repetitions, and instead a comment has been added to refer to the Ethics and Consent declaration for further details.

- ***Literature review***

**Reviewer #1: **The literature review acknowledges sources of evidence between 2011 and 2025 with no rationale for selecting this particular period of time. It would be good to know why this timeframe was decided given that the MSCA was launched in 2014 and its predecessor MCA started in 1996.

**Authors' response:** We have clarified that the literature review’s main focus is on sources published since 2014, corresponding to the two most recent Framework Programmes, Horizon 2020 and Horizon Europe, to provide a recent, policy-relevant overview of the current state of doctoral education. However, some relevant older sources have also been cited in the article depending on their relevance to understanding the state of doctoral education before Horizon 2020, including:

1. 2013 - MORE2: Support for Continued Data Collection and Analysis Concerning Mobility Patterns and Career Paths of Researchers.
2. 2011 - The 1 ^st^ Eurodoc Survey on Doctoral Candidates in Twelve European countries.
3. 2011 - German Rectors’ Conference (HRK): Report of Mapping Exercise on Doctoral Training in Europe: ‘Towards a Common Approach’.
4. 2011 - EC: DG RTD: Principles for Innovative Doctoral Training.
5. 2008 - EC: Communication from the Commission to the Council and the European Parliament: Better Careers and More Mobility: A European Partnership for Researchers.

**Reviewer #1: **There is ambiguity in the first paragraph of section 3.2: “Although historically, there has been a gap in supporting comprehensive skills development among **PhD holders** in…. high standards in **doctoral education**”. Is this referring to postdoctoral training or during doctoral studies or both?

**Authors' response:** No, it is about transferable skills training **during** doctoral studies. The same has been clarified in the revised version.

**Reviewer #1: **The Methodology claims that the literature review was based on “110 existing literature items”, but there is also a statement on Data Availability that reads, “The sources consulted during literature review are provided in the reference section below”; the list displays 49 references, how does this relate to the 110 papers?

**Authors' response:** The 110 sources consulted during initial literature review primarily helped in identifying the key themes and preparing the interview and survey questions. However, the references section in the article only lists the sources that have been cited in the text. The data availability section has been revised accordingly.

**Reviewer #1: **The actual text in the section of the literature review refers to mostly grey literature regarding the programme without acknowledging other empirical studies on MSCA or other kinds of doctoral and postdoctoral training. For instance: [Ref 1 & 2]

**Authors' response:** We had to rely mostly on “grey literature” as comprehensive peer-reviewed study on the MSCA ITN and DN proved to be scarce. However, insights from empirical studies have also been incorporated into the article, wherever available, including the two sources suggested by the reviewer. The article by Doonan et al. was specifically useful during revisions, and has been incorporated in 1. introduction, 3. literature review, 4 findings, and 5. discussions. The article by Nikinmaa and Zacharewicz was useful in supporting that international mobility is a crucial factor influencing career progression of researchers. It has been cited in the beginning of section 3 literature review, where we also clarified that our study shares MSCA’s vision of mobility as part of a broader category of “triple 'i' mobility”, including international, intersectoral, and interdisciplinary. However, since Nikinmaa and Zacharewicz primarily focus on the case of “experienced” researchers in COFUND and IF, and provide no insights on Doctoral Networks, it was left out of the broader engagements in findings and discussions.

**Reviewer #1: **It would be helpful if the reporting of the sources consulted is revised for consistency and coherence purposes.

**Authors' response:** The data availability statement has been revised to reflect that only sources cited directly in the article have been provided in the reference section.

- ***Findings***

**Reviewer #1: **The findings section presents rich data in the text and in relevant Figures. It is unclear whether Figures are based on both the interview and the survey or just the survey. A clarification to this regard should be added, for instance, to the introduction of section 4 or reflected in the Figure captions.

**Authors' response:** A clarification has been added to the introduction of section 4 that all figures are based on the survey, unless explicitly stated otherwise.

**Reviewer #1: **In relation to the above, some statements need precision. For example, the text reads, “As one MSCA supervisor eloquently stated, the intersectoral dimension (and triple “i” mobility as a whole) of MSCA ITN/DN programmes fosters the emergence of excellently trained young scientists, poised to bridge the gap between industry and academia with their diverse skill sets.” There are no quotation marks or indication whether the statement was provided in the interview or the survey.

**Authors' response:** It is from an interview. Quotation marks have been reintroduced, and clarification provided in the revised version.

**Reviewer #1: **The findings offer critical insights with respect to the consistently enhanced mobility reported by those in the programme compared to their peers not participating in it. It would be useful to see more qualitative data in the form of direct or indirect quotes from the participants. This would help support some of the authors’ interpretations of the quantitative data. Also, the current selected quotes reflect positive comments; some of the issues flagged by the participants could be heard as well. One of the exceptions is the identification, analysis and commentary regarding funding awareness.

**Authors' response:** Issues flagged by participants, along with direct and indirect quotes have been added. Please refer to previous responses.

- ***Discussion***

**Reviewer #1: **This section mostly synthesizes the findings, more critical commentary on their significance against the background of previous knowledge would strengthen the argument.

**Authors' response:** Critical commentary on findings against the background of previous knowledge have been added wherever relevant, depending on previous knowledge. The discussion section has been heavily restructured and revised as well. However, as noted in the article, to our knowledge, this is the first comprehensive study on specifically the MSCA ITN and DN’s added values. Therefore, most of our findings are new, with not enough previous data to directly compare against. Furthermore, as the scope of the article is limited to comparing MSCA and non-MSCA doctoral impacts under ITN and DN, the study has abstained from digressing towards existing knowledge on other MSCA actions, or MSCA as a whole.

- ***Recommendations***

**Reviewer #1: **This section is very helpful in practical terms and is supported by the data. There is only a small issue with the initial paragraph which only refers to the survey. This gives the impression of the authors valuing more the quantitative data. Were the interviews insignificant for the recommendations?

**Authors' response:** “The survey results” has been replaced by “the results of the study” as a whole. The recommendations from interviews were indeed equally significant and were in alignment with the quantitative data, complementing each other. Insights from both components have been taken into account in drafting the recommendations.
